# Prevalence and factors associated with polypharmacy: a systematic review and Meta-analysis

**DOI:** 10.1186/s12877-022-03279-x

**Published:** 2022-07-19

**Authors:** Mahin Delara, Lauren Murray, Behnaz Jafari, Anees Bahji, Zahra Goodarzi, Julia Kirkham, Zia Chowdhury, Dallas P. Seitz

**Affiliations:** 1 Department of Psychiatry, Queen’s University, Providence Care-Mental Health Services, Kingston, Ontario Canada; 2 Department of Psychiatry, Hotchkiss Brain Institute, and O’Brien Institute for Public Health, University of Calgary, Calgary, Alberta Canada; 3 Cumming School of Medicine, University of Calgary, Room 2919 Health Sciences Centre, 3330 Hospital Drive NW, Calgary, AB T2N 4N1 Canada; 4 Departments of Medicine and Community Health Sciences, Hotchkiss Brain Institute, and O’Brien Institute for Public Health, Calgary, Alberta Canada

**Keywords:** Polypharmacy, Multiple medication prescribing, Population-based study, Administrative database

## Abstract

**Introduction:**

Polypharmacy is commonly associated with adverse health outcomes. There are currently no meta-analyses of the prevalence of polypharmacy or factors associated with polypharmacy. We aimed to estimate the pooled prevalence of polypharmacy and factors associated with polypharmacy in a systematic review and meta-analysis.

**Methods:**

MEDLINE, EMBASE, and Cochrane databases were searched for studies with no restrictions on date. We included observational studies that reported on the prevalence of polypharmacy among individuals over age 19. Two reviewers extracted study characteristics including polypharmacy definitions, study design, setting, geography, and participant demographics. The risk of bias was assessed using the Newcastle-Ottawa Scales. The main outcome was the prevalence of polypharmacy and factors associated with polypharmacy prevalence. The pooled prevalence estimates of polypharmacy with 95% confidence intervals were determined using random effects meta-analysis. Subgroup analyses were undertaken to evaluate factors associated with polypharmacy such as polypharmacy definitions, study setting, study design and geography. Meta-regression was conducted to assess the associations between polypharmacy prevalence and study year.

**Results:**

106 full-text articles were identified. The pooled estimated prevalence of polypharmacy in the 54 studies reporting on polypharmacy in all medication classes was 37% (95% CI: 31-43%). Differences in polypharmacy prevalence were reported for studies using different numerical thresholds, study setting, and publication year. Sex, study geography, study design and geographical location were not associated with differences in polypharmacy prevalence.

**Discussion:**

Our review highlights that polypharmacy is common particularly among older adults and those in inpatient settings. Clinicians should be aware of populations who have an increased likelihood of experiencing polypharmacy and efforts should be made to review the appropriateness of prescribed medications and occurrence of adverse effects potentially associated with polypharmacy.

**Conclusions and implications:**

Clinicians should be aware of the common occurrence of polypharmacy and undertake efforts to minimize inappropriate polypharmacy whenever possible.

**Supplementary Information:**

The online version contains supplementary material available at 10.1186/s12877-022-03279-x.

## Introduction

While medications are often necessary to manage acute and chronic health conditions, polypharmacy can be a significant problem related to prescribed medications. Polypharmacy refers to a situation where an individual uses multiple medications simultaneously. The World Health Organization (WHO) defines polypharmacy as “the administration of many drugs at the same time or the administration of an excessive number of drugs“ [1]. While there is no consensus on the medication threshold and means of measurement, polypharmacy is often commonly defined as concomitant use of 5 or more medications [1, 2].

Prescribing multiple medications is often clinically required (appropriate polypharmacy). However, exposure to multiple medicines may lead to harm or the ongoing use of medications no longer indicated (inappropriate polypharmacy) [2, 3]. Polypharmacy can be associated with numerous poor health outcomes, especially among older adults with multimorbidity, including an increased risk of death, falls, drug interactions, non-adherence, and hospitalization [2, 3]. Polypharmacy has become a substantial health care burden. It is associated with an annual estimated cost of $50 billion US, which is increasing over time [4]. To avoid such costs and potentially prevent adverse events associated with polypharmacy, identification of individuals who are at high risk of receiving inappropriate polypharmacy is an important first step [3].

Some research indicates that inappropriate polypharmacy can affect up to one-third of populations [4]. However, an accurate estimate of the prevalence of polypharmacy requires the incorporation of information from multiple studies. Also, the factors associated with polypharmacy such as patient and healthcare characteristics have not been well-described. We conducted this systematic review and meta-analysis of population-based observational studies to estimate the prevalence of polypharmacy and identify factors associated with polypharmacy.

## Methods

We registered our review protocol on PROSPERO (CRD42019130998). We followed the Preferred Reporting Items for Systematic Reviews and Meta-analysis (PRISMA) guidelines for our systematic review [5].

### Study inclusion criteria

We included population-based observational studies including all cross-sectional, case-control, or cohort designs using administrative databases or registries to define or measure polypharmacy among individuals over 19. We excluded studies that focused on children and adolescents (aged 0-18) as use of prescribed medications is relatively uncommon in these age groups. We included all publication dates and limited articles to the English language only. The primary study objective outcome was to estimate polypharmacy prevalence and identify factors associated with polypharmacy. We also identified definitions and measurements of polypharmacy using administrative databases or registries.

### Search strategy and study selection

We developed a search strategy in consultation with a librarian specializing in health databases. We searched EMBASE, MEDLINE, and Cochrane Library from inception to March 30, 2019, using individualized search strategies prepared for each database. We limited results to studies conducted in humans and available in the English language. We used MeSH terms (e.g. polypharmacy, polytherapy, poly medication, poly prescription, multi medication, multi prescription, multidrug therapy, multiple drug treatment, multiple pharmacotherapies, administrative data, databases, registries) and combinations of relevant keywords and their variants by grouping polypharmacy and administrative database associated terms. The MEDLINE search strategy is presented in Additional file 1 and this was adapted for the other databases*.* We also hand-searched for additional publications using a reference list of relevant papers. Reference management and citation screening were performed using EndNote™ (V.X7) [6]. Using a two-step process for study selection, two of three reviewers (MD, LM) independently screened each citation’s title and abstract to determine whether a study met the inclusion criteria. The full texts of all relevant citations were retrieved for formal review. Two reviewers (MD, LM) independently assessed the full-text reports. Conflicts were resolved by discussion between reviewers.

### Data extraction and management

We created a standardized data extraction form and piloted and modified the form for our review. Two reviewers (MD, LM) extracted data independently, using this form, with conflicts resolved through consensus. The extracted study characteristics included study identifiers, study country, year of publication, sample size, setting, study design, and age and sex of participants. The prevalence of polypharmacy was extracted from each study using the numerator and denominator of the number of participants meeting polypharmacy criteria and the total study sample. Information on the definitions of polypharmacy used in individual studies were recorded including the medication cut-offs used to define polypharmacy, the time period of polypharmacy assessment, the number of overlapping days, and terminology used to define polypharmacy.

### Quality assessment

We assessed the risk of bias and quality of the included studies using the Newcastle-Ottawa Scales for cross-sectional, cohort and case control studies [7]. These tools consist of 7 to 8 domains depending on the type of study design; each domain is rated ‘low risk,’ ‘unclear risk,’ or ‘high risk.’ Studies were categorized based on total scores as being of either high quality (total scores ≥7) or low quality (total score < 7).

### Data analysis

The included studies’ data were summarized in frequency tables and analyzed using RStudio (version 3.6.1). Meta-analyses were performed using random effects models using inverse variance weighting. Meta-analysis was used to estimate of the pooled prevalence of polypharmacy and 95% confidence intervals (CI). Heterogeneity was determined using the I-squared (I^2^) statistic and categorized as low < 25%, moderate 25–50%, high > 50% heterogeneity [8]. If significant heterogeneity was suspected, further analysis, including subgroup analysis, was conducted to explore potential sources of heterogeneity. We used meta-regression analysis to assess the associations between polypharmacy prevalence and publication year. Subgroup analysis of age was performed using either the mean age of the study population or the median age of the study population depending on which measure of age was reported in individual studies and we categorized age as < 65 years or ≥ 65 years. Publication bias was assessed for the prevalence of polypharmacy using funnel plots and Egger’s test [9].

### Subgroup analyses

We restricted subgroup analyses to situations where a minimum of 4 studies were available for each categorical study-level variable [10]. A total of nine subgroup analyses were planned based on: mean age (< 65 vs ≥ 65 years); sex of participants (male vs female), geography of study (Europe, Asia, North America, Australia, South America, Africa), health care setting (inpatient vs outpatient vs community), study design (cross-sectional vs case-control vs cohort), methodological quality (low vs high); medication threshold (2 vs 5), measurement indicator (simultaneous vs cumulative).

## Results

**Fig. 1:**
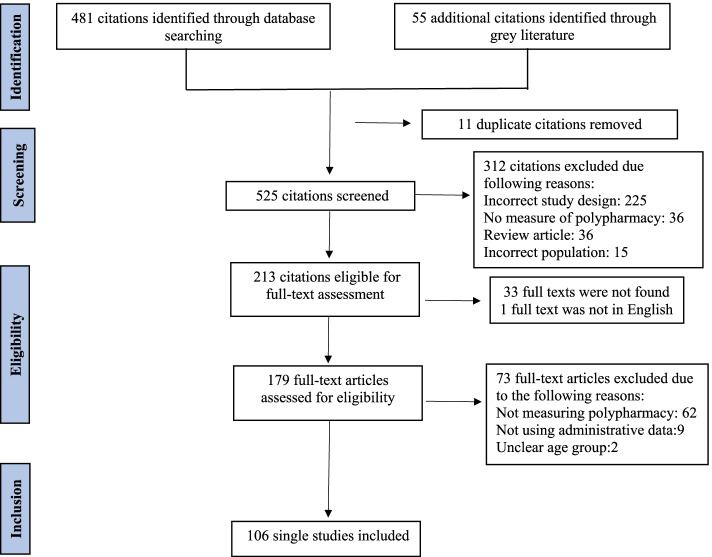
Flow diagram for study selection

Of 525 unique citations, we retrieved and reviewed 179 full text articles. Of these full text articles, 106 studies met inclusion criteria [1, 11–115]. The studies were published from 1989 to 2019. Figure 1 depicts a PRISMA flowchart of citations reviewed. The baseline characteristics of studies are summarized in Additional File 2. While two studies were multicentre, most studies were conducted in Europe (*n* = 59), followed by Asia (*n* = 21), North America (*n* = 20), Australia (*n =* 2), and South America (*n =* 2). The healthcare setting was specified in 50 studies (49.1%) including community (*n* = 15), inpatient (*n =* 15), outpatient (*n* = 13) or a combination of these settings (*n* = 7). Nearly 75% percent of the studies were cross-sectional (*n* = 80). The studies included a different number of participants varying from 248 to 12,301,537 individuals. The mean age of participants ranged from 26 to 87 years old.

### Definitions of polypharmacy

Fifty-seven studies used the term polypharmacy (*n* = 57, 54%) followed by polytherapy (*n* = 18 studies, 17%), co-prescribing (2 studies,1.9%), multiple medication use (1 study, 0.9%), concurrent medication (1 study, 0.9%), poly medication (1 study, 0.9%), concomitant use (1 study, 0.9%) and comedication (1 study, 0.9%) ***(***Additional File 3***)***. Out of the 106 identified articles, 96 (90.6%) included information on the number of medications required for a definition of polypharmacy resulting in 124 different definitions with the remaining studies not reporting a specific medication threshold. Among studies reporting a medication threshold the threshold for polypharmacy varied between 2 and 21 medications. A medication threshold of ≥5 medications was the most commonly used threshold (52 studies, 49%) followed by a threshold of ≥2 medications (39 studies, 37%). All descriptive definitions had a time component, including the duration of therapy, minimum days of overlap, time interval, or assessment frequency. Three studies expressly incorporated an allowable gap of 12, 14, and 15 days in their definitions, and one study reported a grace period of 28 days.

### Measuring polypharmacy

The definitions used for polypharmacy varied across studies with 59 studies using a simultaneous approach (55.7%), 45 using the cumulative method (42.5%), and two studies examined a combination of simultaneous and cumulative approaches (1.9%) (Additional file 3). Studies using a simultaneous definition of polypharmacy required 2 or more medications to be prescribed concurrently. The most common duration of concurrent use was described in studies using the concurrent approach was at least 1 day overlap between medications (47 studies, 44%). The minimum overlap days required for the simultaneous approach ranged from 1 day to 1 year. Cumulative definitions assessed the number of unique medications prescribed at any time during a specified time period without requiring the medications to be overlapping for the entire time period. Among studies using a cumulative approach, a 90-day time period was identified as the most duration in which cumulative medications were prescribed (9 studies, 9%). The timing of polypharmacy assessment also differed across studies. Studies using the cumulative approach specified a period between 14 to 730 days.

### Prevalence of polypharmacy

#### Overall prevalence

**Fig. 2:**
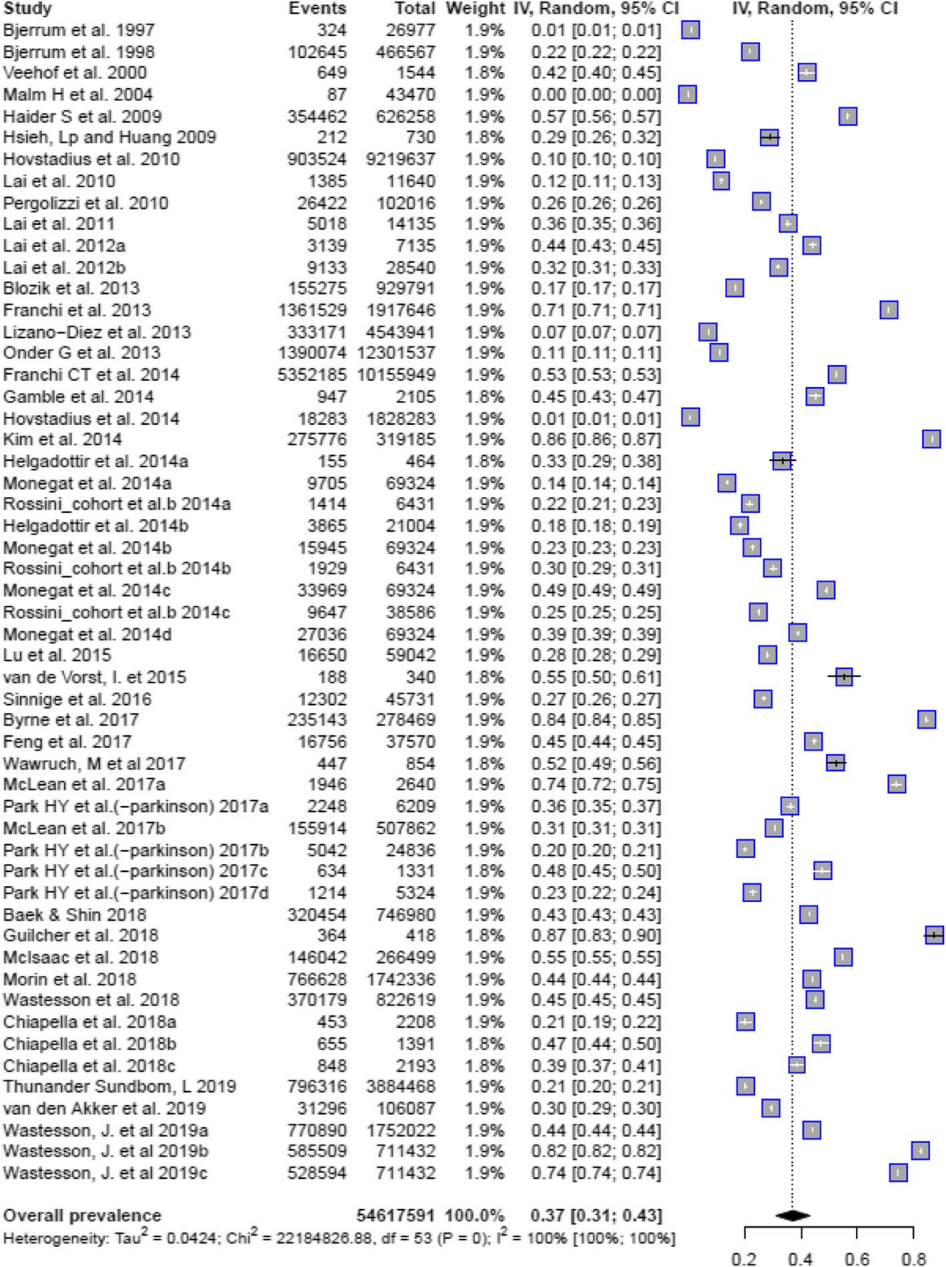
Prevalence of polypharmacy across included studies

Nighty-four studies (100 single reports) contained sufficient quantitative information to determine the prevalence of polypharmacy. Of the 94 studies, 54 reported on polypharmacy incorporating all medications. The remaining studies only reported polypharmacy related to specific medication classes (e.g. psychotropic polypharmacy, polypharmacy with antiepileptic medications). The pooled estimated prevalence of polypharmacy among the 54 studies reporting on polypharmacy including all medications was 37% (95% CI: 31 - 43%). There was a high degree of heterogeneity observed in this estimate (Tau^2^=0.0424, Chi^2^=22,194,826, df = 53, I^2^ = 100%) **(**Fig. 2**).** Due to the heterogeneity in the definitions of polypharmacy used in other studies we did not include studies that did not report on the prevalence of polypharmacy using all medication classes.

#### Factors associated with polypharmacy

Subgroup analyses were undertaken using the studies reporting polypharmacy including all medication classes. These subgroup analyses included sex, numerical threshold (≥2 medications vs ≥5 medications), study geography (North America vs other), study design (cross-sectional, case-control, or cohort) and study setting (community, outpatient and inpatient) or study location (North America vs outside North America). Meta-regression was used to assess the associations between polypharmacy and study year. A summary of the results of the subgroup analyses are provided in Table 1.

**Table 1:** Summary of Subgroup Analyses of Polypharmacy Prevalence

Subgroup	Pooled Prevalence (95% CI)	Chi^**2**^	df	***p***-value
Mean age of participants	36 (29,43)	10.88	1	< 0.01
< 65 years	25 (16,35)	238,592.68	20	< 0.0001
≥ 65 years	45 (37,54)	4,378,199.84	25	< 0.0001
Sex	32 (26,39)	0.04	1	0. 85
Male	32 (22,41)	1,484,546.49	21	< 0.0001
Female	33 (23,43)	1,754,816.42	21	< 0.0001
Health care setting	40 (32,49)	13.55	2	< 0.01
Inpatient	52 (38,66)	36,809.71	10	< 0.0001
Outpatient	47 (33,62)	910,219.51	12	< 0.0001
Community	20 (6,35)	12,462,667.1	10	< 0.0001
Numerical cut off for medications	32 (26,38)	7.90	1	< 0.01
≥ 2 medications	22 (10,35)	22,184,826.88	9	< 0.0001
≥ 5 medications	40 (0, 47)	16,672,729.06	43	< 0.0001
Measurement indicator	33 (28,38)	0.01	2	*P* = 0.91
Simultaneous	33 (27,40)	9,353,584.02	50	< 0.0001
Cumulative	33 (25,41)	12,926,693.67	37	< 0.0001
Methodological quality	33 (28,38)	0.37	1	0.54
High quality	34 (29.39)	21,220,449.4	83	< 0.0001
Low quality	27 (0,57)	5005.5	4	< 0.0001
Country of publication	37 (31,44)	2.19	2	0.33
North America	51 (24,79)	30372.7	4	< 0.0001
Asia	36 (24,48)	377564.9	11	< 0.0001
Europe	36 (27,44)	20277561.9	33	<0.001
Study design	33 (28,38)	9.93	2	0.31
Cross-sectional	37 (31,43)	20,427,507.22	63	< 0.0001
Cohort	23 (9,38)	326,083.41	11	< 0.0001
Case-control	24 (17,31)	365,802.26	12	< 0.0001

Chi^2^ = Chi-squared; df = degrees of freedom; Number of reports exceeding the total number of included studies indicates that some studies contributed more raw data at different study periods, age groups or etc.

Subgroup analysis by age indicated that studies with a population age of ≥65 years were associated with a higher prevalence of polypharmacy (45, 95% CI: 37 to 54%) when compared to studies with population age of < 65 years (25, 95% CI: 15 - 35%, *P* < 0.01, Additional file 4). Studies using a threshold of ≥2 medications had a lower reported prevalence of polypharmacy (22, 95% CI: 10 - 35%, *N* = 9) when compared to studies using a threshold of ≥5 medications (40, 95% CI: 0 – 47%; *N* = 43; P < 0.01, Additional file 5***)***. There was also a significant difference in the estimates of polypharmacy between health care settings with lower estimates of polypharmacy associated with community settings (20, 95% CI: 6 - 35%) when compared to outpatient (37, 95% CI: 33 - 66%) and hospital settings (52, 95% CI: 38 - 66%, Additional file 6). There were no differences in polypharmacy prevalence in subgroup analyses based on sex (Additional file 7), or geographical location (Additional file 8***)*** or study design (Additional file 9).

Meta-regression demonstrated that more recent studies were associated with a higher prevalence of polypharmacy (β estimate = 0.0175, *p* = 0.003).

### Assessment of study quality

Studies were overall adjudicated to be at low risk of bias or high quality (101 studies, 95.3%), and five studies were judged to be at high risk of bias or low quality. The assessment of study quality according to the Newcastle-Ottawa Scale risk of bias assessments for cohort, case-control and cross-sectional studies are provided in Additional file 10. Overall, 10/13 cohort (77%), 11/13 of case-control (85%) and 80/80 cross-sectional studies (100%) were reported to be of high quality.

### Publication Bias assessment

The Egger’s test for publication bias did not indicate any potential evidence of publication bias (t = 2.06, d.f. 52, *P* = 0.05) and the funnel plot is presented in Additional File 11.

## Discussion

Overall, our review indicates that polypharmacy is common with an estimated overall prevalence of 37%. Older age, inpatient clinical settings and more recent studies were associated with a higher prevalence of polypharmacy. Our review also identified several definitions used to define polypharmacy in the literature with the term polypharmacy and a threshold of ≥5 medications were the most common definitions found in the literature. While our review could not incorporate contextual information related to comorbid medical conditions into our assessment of polypharmacy, this information is also necessary to understanding if polypharmacy is appropriate or inappropriate not for an individual which is critical to optimizing prescribing of medications and balancing potential benefits and risks associated with the prescribing of multiple medications.

Our review provides new information about patient populations at high risk for experiencing polypharmacy particularly older adults and those in outpatient or hospital inpatient settings. Clinicians should be aware of the high prevalence of polypharmacy particularly in these contexts and consider implementing processes to review medications for their appropriateness to reduce potential adverse effects from inappropriate polypharmacy. Awareness of polypharmacy is important as it is associated with several adverse outcomes such as the increased risk of drug-drug interactions, hospitalizations, functional decline and mortality [116–119]. When the number of prescribed medicines increases, the number of drug combinations increases exponentially, increasing the risk of adverse drug reactions and drug- drug interactions [120]. Whether polypharmacy is directly causing these outcomes or if it is a marker for frailty or general vulnerability to poor outcomes is not always clear. However, the presence of polypharmacy may be an indicator for clinicians to identify individuals at risk for adverse outcomes who may benefit from preventative health measures and medication review.

Our review identified several different terms and thresholds are used to define polypharmacy consistent with a related review on this topic [3]. Masnoon et al. [3] identified 110 articles that assessed polypharmacy. However, these authors highlighted the diverse range of terms used to define polypharmacy, such as minor, major, severe, excessive, hyper, appropriate, persistent, chronic, long term, and pseudo-polypharmacy [3]. Our review identified that different medication thresholds may impact on the estimates of polypharmacy. While we did not observe any difference in the estimated prevalence of polypharmacy using continuous or simultaneous approaches to defining polypharmacy it has been suggested that different methods of measuring polypharmacy may have different clinical implications [1]. Simultaneous assessments of polypharmacy may be more helpful for exploring outcomes potentially adverse events directly related to drug-drug interactions as simultaneous polypharmacy assesses concurrent use of medications at the same time. Cumulative measures of polypharmacy may help identify potential risks associated with polypharmacy during a period of time which may also be associated with adverse outcomes or reflect changes to individual’s clinical status over time. Both our review and this previous review highlight the variety of terms used to define polypharmacy and emphasize the need for researchers to employ standardized definitions of polypharmacy in the future including incorporating information about inappropriate polypharmacy. Further study is also required to understand if different definitions of polypharmacy are associated with different risks of adverse outcomes.

Identifying any polypharmacy is the first step towards assessing whether polypharmacy is inappropriate. Several strategies can be employed to reduce inappropriate prescribing or inappropriate polypharmacy. Medication review (by pharmacists, physicians, or multidisciplinary teams), education and training, and the use of screening tools to identify potentially inappropriate prescribing (e.g. Screening Tool of Older Persons’ Potentially Inappropriate Prescription (STOPP)) have all been found to be effective for reducing polypharmacy in various populations [121–126]. Digital technologies (for example, automatically generated alerts in electronic prescribing programs) have shown promising results in lowering polypharmacy in various settings but have not been widely adopted or investigated [126]. Most of these studies focus on interventions to reduce polypharmacy in settings of greatest concern, such as among older adults or those in residential care settings. However, interventions to reduce polypharmacy vary widely, and the most effective aspects of interventions are still unclear [125]. Deprescribing does not appear to increase adverse outcomes, [127] but whether reducing polypharmacy results in improved outcomes is not as clear. Studies incorporating clinical outcomes have had mixed findings on the effects of deprescribing on the quality of life, falls, disease-specific outcomes, and hospitalizations [122]. Several reviews of interventions to reduce polypharmacy found no effect on all-cause mortality [121, 123, 127].

Polypharmacy is a complex issue and may differ in appropriateness and implications for medically complex individuals compared to those who are healthier. In general, polypharmacy needs to be justified and limited as much as possible, especially in older or frail adults. Several strategies have been proposed to reduce polypharmacy, such as deprescribing, reducing the use of unnecessary and inappropriate drugs, and underuse of medications. Still, their clinical significance is not well known [120]. One of the main challenges is to disentangle the effects of removing drugs from reducing the overall burden of medicines that are not causing harm or adverse events [120]. As well, the use of polypharmacy definitions in clinical practice and informatics systems depends on their operationalization and utility [1]. Therefore, the best approaches to addressing polypharmacy in different contexts and populations requires further consideration.

Our study has several strengths. By conducting a meta-analysis of the prevalence of polypharmacy which helps provide a better understanding of the frequency of polypharmacy. Our review also highlights important factors associated with the prevalence of polypharmacy which previously had primarily been described in individual studies. Our search strategy identified over 100 studies reporting on polypharmacy which will help produce less biased estimates of the prevalence of polypharmacy. Our review also identified that there is a relatively high quality of evidence on this topic, as the majority of studies were at low risk of bias. Despite these strengths, we acknowledge that this work has some limitations. Most studies operationalized polypharmacy as multiple medication use, so we could not distinguish between appropriate and inappropriate polypharmacy. The prevalence estimates in this review were based mainly on dispensing data and adherence to prescribed medications was not available in studies. The included studies did not use a homogenous study populations or polypharmacy which contributed to heterogeneity observed across studies. While the observed difference among some subgroups was not significant, the high heterogeneity within those subgroups may reflect the presence of unmeasured factors influencing heterogeneity such as comorbidities, prescription, adherence, medication dosage or other factors which may also influence estimates of polypharmacy. We also only included studies that used administrative or registry data which may have excluded some studies on polypharmacy which used alternative sources of data.

## Conclusion and implications

Our study highlights that polypharmacy is common among adults particularly among older adults and those in inpatient settings. Given the adverse outcomes potentially associated with polypharmacy, it is important to understand both the prevalence of polypharmacy and those populations at highest risk for being exposed to polypharmacy. Clinicians should regularly assess patients for the presence of polypharmacy and institute measures to reduce inappropriate polypharmacy when it is possible to do so. Efforts should continue to be made to harmonize definitions of polypharmacy to facilitate more consistent reporting of polypharmacy in the research literature and information on the appropriateness of polypharmacy should be incorporated into reporting of polypharmacy in future studies.

## Supplementary Information

**Additional file 1.** Search Strategy using Medline Database.

**Additional file 2.** Baseline Characteristics of Included Studies.

**Additional File 3.** Descriptive Definitions and Measurement Indicators of Polypharmacy and Alternative Terms across Studies.

**Additional file 4.** Prevalence of Polypharmacy Associated with Different Age Subgroups.

**Additional file 5.** Prevalence of Polypharmacy Associated with Different Medication Thresholds.

**Additional file 6.** Prevalence of Polypharmacy Associated with Settings of Study.

**Additional file 7.** Prevalence of Polypharmacy Associated with Sex.

**Additional file 8.** Prevalence of Polypharmacy Associated with Study Geographical Location.

**Additional file 9.** Prevalence of Polypharmacy Associated with Different Study Designs.

**Additional file 10. **Risk of Bias Summary in Included Studies (*n* = 106).

**Additional file 11.** Funnel Plot of Prevalence of Polypharmacy in Included Studies.

## Data Availability

The datasets used and/or analysed during the current study are available from the corresponding author on reasonable request.

## Abbreviations

CI: Confidence Interval
PRISMA: Preferred Reporting Items for Systematic Reviews and Meta-analysis
STOPP: Screening Tool of Older Persons’ Potentially Inappropriate Prescription
