# Correction: Prevalence and factors associated with polypharmacy: a systematic review and meta-analysis

**DOI:** 10.1186/s12877-022-03388-7

**Published:** 2022-09-12

**Authors:** Mahin Delara, Lauren Murray, Behnaz Jafari, Anees Bahji, Zahra Goodarzi, Julia Kirkham, Mohammad Chowdhury, Dallas P. Seitz

**Affiliations:** 1 Department of Psychiatry, Queen’s University, Providence Care-Mental Health Services, Kingston, Ontario Canada; 2 Department of Psychiatry, Hotchkiss Brain Institute, and O’Brien Institute for Public Health, University of Calgary, Calgary, Alberta Canada; 3 Cumming School of Medicine, University of Calgary, Room 2919 Health Sciences Centre, 3330 Hospital Drive NW, Calgary, AB T2N 4N1 Canada; 4 Departments of Medicine and Community Health Sciences, Hotchkiss Brain Institute, and O’Brien Institute for Public Health, Calgary, Alberta Canada

**Correction: BMC Geriatr 22, 601 (2022)**

**https://doi.org/10.1186/s12877-022-03279-x**

After publication of this article [1], the authors reported that the author name ‘Mohammad Chowdhury’ was incorrectly written as ‘Zia Chowdhury’.

In addition, the last author (Dallas P. Seitz) is affiliated to affiliations 3 and 4 (instead of affiliation 1).

The original article [1] has been corrected.
