# Supplementary material for: Prevalence and factors associated with polypharmacy: a systematic review and Meta-analysis
Source: BMC Geriatr. 2022 Jul 19;22:601. doi: 10.1186/s12877-022-03279-x (PMC9297624; doi:10.1186/s12877-022-03279-x)
Supplement: Supplementary file 1 — Additional file 1. Search Strategy using Medline Database. [file 12877_2022_3279_MOESM1_ESM.docx]

## Additional file 1. Search Strategy using Medline Database

| 1 | polypharmacy*.mp. | 10699 |
| --- | --- | --- |
| 2 | multi-drug therapy*.mp. | 357 |
| 3 | multidrug therapy*.mp. | 1270 |
| 4 | multidrug therapy*.mp. | 1270 |
| 5 | multiple drug therapy*.mp. | 430 |
| 6 | multiple drug treatment*.mp. | 109 |
| 7 | multiple pharmacotherapy*.mp. | 8 |
| 8 | pharmacotherapy,multiple*.mp. | 3 |
| 9 | pharmacy, poly*.mp. | 1 |
| 10 | polypragmasia*.mp. | 37 |
| 11 | polypragmas*.mp. | 106 |
| 12 | polytherapy*.mp. | 1838 |
| 13 | poly medication*.mp. | 18 |
| 14 | poly prescription*.mp. | 5 |
| 15 | multi medication*.mp. | 23 |
| 16 | multi prescription*.mp. | 4 |
| 17 | multidrug therapy*.mp. | 1270 |
| 18 | multiple drug treatment*.mp. | 109 |
| 19 | multiple pharmacotherapies*.mp. | 17 |
| 20 | concomitant pharmacotherapy*.mp. | 45 |
| 21 | combined pharmacotherapy*.mp. | 177 |
| 22 | polydrug*.mp. | 1617 |
| 23 | drug co-administered*.mp. | 4 |
| 24 | drug combinations*.mp. | 80552 |
| 25 | 1 or 2 or 3 or 4 or 5 or 6 or 7 or 8 or 9 or 10 or 11 or 12 or 13 or 14 or 15 or 16 or 17 or 18 or 19 or 20 or 21 or 22 or 23 or 24 | 96688 |
| 26 | administrative data*.mp. | 14368 |
| 27 | (registries* or registry*).mp. | 179765 |
| 28 | 26 or 27 | 192761 |
| 29 | 25 and 28 | 808 |
| 30 | limit 29 to (full text and human and English language) | 235 |
