# Supplementary material for: Prevalence and factors associated with polypharmacy: a systematic review and Meta-analysis
Source: BMC Geriatr. 2022 Jul 19;22:601. doi: 10.1186/s12877-022-03279-x (PMC9297624; doi:10.1186/s12877-022-03279-x)
Supplement: Supplementary file 2 — Additional file 2. Baseline Characteristics of Included Studies. [file 12877_2022_3279_MOESM2_ESM.docx]

## Additional file 2. Baseline Characteristics of Included Studies

| **Authors** | **Country** | **Setting** | **Design** | ***N*** | **Female (%)** | **Age** | **NOS** |
| --- | --- | --- | --- | --- | --- | --- | --- |
| Källén et al. (1989) | Multicenter* | - | Cross-Sectional | 377 | 100 | - | 8 |
| Bjerrum et al (1997) | Denmark | - | Cross-Sectional | 26977 | - | - | 8 |
| Bjerrum et al. (1998) | Denmark | - | Cross-Sectional | 466567 | - | Minimum:16 | 8 |
| Veehof et al. (2000) | Netherlands | - | Cross-Sectional | 1544 | 61 | Mean: 73; Minimum: 65 | 10 |
| Weissman (2002) | USA | - | Cross-Sectional | 2987 | - | All age groups | 8 |
| Hamann et al. (2003) | Germany | Outpatient | Cross-Sectional | 671454 | - | Minimum:18 | 7 |
| Jaffe & Levine (2003) | USA | Inpatient | Cross-Sectional | 8212 | 34.4 | Mean (SD): 45 (13) | 8 |
| De las Cuevas & Sanz (2004) | Spain | Community | Cross-Sectional | 2647 | 71.1 | Mean (SD): 50(17); Range: 15-90 | 9 |
| Malm et al. (2004) | Finland | - | Cohort | 86940 | 100 | - | 7 |
| French et al. (2005) | USA | Outpatient | Case-Control | 6636 | 3 | Mean: 74, Median: 77, Range: 30-101 | 4 |
| Preskorn et al. (2005) | USA | Outpatient | Cross-Sectional | 5003 | 4.3 | Mean (SD): 63 (13) | 9 |
| Åstrand et al. (2006) | Sweden | Community | Cross-Sectional | 8214 | 57.8 | Mean (SD): 50 et al. (23) | 9 |
| Åstrand et al. (2007) | Sweden | Community | Cross-Sectional | 8214 | 57.8 | Mean (SD): 48 et al. (24) | 9 |
| Targownik et al. (2007) | Canada | - | Cross-Sectional | 887165 | - | Mean: 64; Minimum: 18 | 10 |
| Carey et al. (2008) | UK | - | Cross-Sectional | 218567 | 57 | Minimum:65 | 10 |
| Dolk et al. (2008) | Netherlands | - | Case-Control | 85563 | 100 | Mean:29 | 6 |
| Gidal et al. (2009) | USA | - | Cross-Sectional | 11206 | 61 | Minimum:18 | 8 |
| Haider et al. (2009) | Sweden | - | Cross-Sectional | 626258 | 60 | Mean:81; Range:75-89 | 10 |
| Hsieh & Huang (2009) | Taiwan | - | Cross-Sectional | 730 | 45.6 | All age groups | 9 |
| Hovstadius et al. (2010) | Sweden | Community | Cross-Sectional | 9219637 | - | All age groups | 8 |
| Lai et al. (2010) | Taiwan | Outpatient | Case-Control | 11640 | 52.2 | Minimum:65 | 7 |
| Moisan & Grégoire (2010) | Canada | Outpatient | Cohort | 54.6 | 46074 | Range: 20-64 | 7 |
| Slabaugh et al. (2010) | Italy | - | Cross-Sectional | 537387 | 58.3 | Mean (SD): 76 (8) | 9 |
| Charlton et al. (2011) | UK | - | Cohort | 2019 | 100 | Mean (SD): 30 (6); Range: 14-49 | 7 |
| Hoffmann et al. (2011) | Germany | Outpatient | Cross-Sectional | 1848 | 47.6 | Mean:79 | 10 |
| Kragh et al. (2011) | Sweden | Inpatient | Cross-Sectional | 2043 | 96.2 | Mean (SD): 83 (8) | 8 |
| Kulaga et al. (2011) | Canada | - | Cohort | 349 | 100 | Mean (SD): 27 (6); Range: 15-45 | 7 |
| Lai et al. (2011) | Taiwan | Outpatient | Case-Control | 14135 | 48.2 | Minimum:65 | 8 |
| Landmark et al. (2011) | Norway | - | Cross-Sectional | 44611 | - | Range: 0-102 | 9 |
| Pergolizzi Jr et al. (2011) | USA | ≥ 1 setting | Cross-Sectional | 102016 | 66.8 | Minimum:21 | 10 |
| Sanglier et al. (2011) | USA | - | Cohort | - | 70.5 | Mean (SD): 61 (9) | 7 |
| Andrew et al. 2012) | UK | - | Cross-Sectional | 576 | 59.3 | Mean: 38 | 9 |
| Baandrup et al. (2012) | Denmark | Outpatient | Cross-Sectional | 658 | 42 | Range:18-64 | 9 |
| Lai et al. (2012) | Taiwan | - | Case-Control | 35675 | 48 | Minimum:65 | 7 |
| Tiihonen et al. (2012) | Finland | - | Cross-Sectional | 2588 | 38 | Mean (SD):38 (14); Range:16-65 | 10 |
| Xiang et al. (2012) | Multicenter † | Inpatient | Cross-Sectional | 1439 | 47.7 | Mean (SD): 63 (7); Minimum: 55 | 9 |
| Blozik et al. (2013) | Switzerland | Community | Cross-Sectional | 929791 | 52 | Minimum:18 | 9 |
| Calderón-Larrañaga et al. (2013) | Spain | - | Cross-Sectional | 79089 | 55.3 | Mean:47 | 9 |
| Curkendall et al. (2013) | USA | - | Cross-Sectional | 117702 | 45.4 | Mean:57 | 9 |
| Franchi et al. (2013) | Italy | Community | Cross-Sectional | 1917646 | 50.24 | Range: 65-94 | 8 |
| Gören et al. (2013) | USA | - | Cross-Sectional | 933 | 7 | Mean (SD): 51 (12) | 9 |
| Lizano-Díez et al. (2013) | Spain | Community | Cross-Sectional | 5105551 | 48.4 | Mean (SD): 75(11); Minimum: 5 | 9 |
| Onishi et al. (2013) | Japan | ≥ 1 setting | Cross-Sectional | 7338 | 49.8 | Mean (SD): 37 (11) | 8 |
| Palmsten et al. (2013) | USA | - | Cohort | 100942 | 100 | Medians range: 23-27 | 7 |
| Ruwald et al. (2013) | Denmark | - | Cross-Sectional | - | 50.2 | Minimum:50 | 10 |
| Suokas et al. (2013) | Finland | Inpatient | Cross-Sectional | 45731 | 45 | Mean:48 | 9 |
| Wong et al. (2013) | China | - | Cross-Sectional | 223287 | 54.8 | Mean (SD): 60 (18) | 8 |
| Degli Esposti et al. (2014) | Italy | - | Cross-Sectional | 12943 | 48.1 | Mean: 50 | 8 |
| Fano et al. (2014) | Italy | - | Cross-Sectional | 331923 | 54.5 | Minimum:35 | 10 |
| Fereshtehnejad et al. (2014) | Sweden | - | Cross-Sectional | 5907 | 65.4 | Mean (SD): 77 (8) | 9 |
| Franchi et al. (2014) | Italy | Community | Cross-Sectional | 10155949 | 57.7 | Range: 65-94 | 8 |
| Frandsen et al. (2014) | Denmark | - | Case-Control | 37256 | 100 | Minimum:20 | 7 |
| Gamble et al. (2014) | Canada | ≥ 1 setting | Cross-Sectional | 2105 | 50 | Mean (SD): 78 (8) | 9 |
| Guidoni et al. (2014) | Brazil | Inpatient | Cross-Sectional | 3048 | 53.2 | Mean (SD): 56 (19) | 9 |
| Helgadóttir et al. (2014) | Sweden | Inpatient | Case-Control | 21747 | 51.2 | Minimum:65 | 7 |
| Hovstadius et al. (2014) | Sweden | Community | Cross-Sectional | 1828283 | - | Range:65-100 | 8 |
| Kim et al. (2014) | South Korea | Outpatient | Cross-Sectional | 319185 | 60 | Minimum:65 | 10 |
| Monégat et al. (2014) | France | - | Cross-Sectional | - | 69324 | Minimum: 75 | 9 |
| Onder et al. (2014) | Italy |  | Cross-Sectional | 12301537 | - | Minimum: 65 | 9 |
| Pottegård et al. (2014) | Denmark |  | Cross-Sectional | 146959 | 56 | Minimum:18 | 9 |
| Rossini et al. (2014) | Italy | Inpatient | Cohort | 45017 | 100 | Mean (SD): 81 (7) | 8 |
| Wang et al. (2014) | Taiwan | - | Cross-Sectional | 1000000 | - | Minimum:9 | 9 |
| Gaviria et al. (2015) | Spain | Outpatient | Cross-Sectional | 1765 | 31.6 | Mean (SD): 44 (14) | 10 |
| Laflamme et al. (2015) | Sweden | Community | Case-Control | 321995 | 66 | Minimum:65 | 8 |
| Lin et al. (2015) | Taiwan | - | Cross-Sectional | 18141 | 49.6 | Mean (SD): 59 (20) | 9 |
| Lu et al. (2015) | Taiwan | Outpatient | Cross-Sectional | 59042 | 48.8 | Range: 65-74 | 10 |
| Salahudeen et al. (2015) | New Zealand | - | Cross-Sectional | - | 54.9 | Mean (SD): 75 (8) | 10 |
| van de Vorst et al. (2015) | Netherlands | - | Cross-Sectional | 340 | 58.2 | Mean: 80 (IQR: 74-84); Minimum: 40 | 9 |
| Baandrup et al. (2016) | Denmark | Community | Cross-Sectional | 71254 | 53.4 | - | 9 |
| Chang et al. (2016) | Taiwan | - | Cross-Sectional | 2703 | 51.8 | Mean (SD): 40 (19) | 9 |
| Ekstam & Elmståhl (2016) | Sweden | Inpatient | Cohort | 2043 | 76.3 | Mean (SD): 83(8) | 7 |
| Horváth et al. (2016) | Hungary | - | Cross-Sectional | 1282 | 52.6 | Mean (SD):48 (18) | 10 |
| Sinnige et al. (2016) | Netherlands | - | Cross-Sectional | 28595 | 54.1 | Minimum:55 | 10 |
| van den Bemt et al. (2016) | Netherlands | Inpatient | Cross-Sectional | 249 | 45.4 | Mean (SD): 44 (18) | 9 |
| van Erning et al. (2016) | Netherlands | - | Case-Control | 5470 | 50 | Mean (SD):78 (5) | 9 |
| Abe et al. (2017) | Japan | - | Cross-Sectional | 473,487 | - | All age groups | 8 |
| Broeks et al. (2017) | Denmark | - | Cross-Sectional | 336 | 100 | Range: 15-55; Median: 30 | 8 |
| Byrne et al. (2017) | Ireland | Community | Cross-Sectional | 278469 | 54.3 | Minimum:16 | 10 |
| Caughey et al. (2017) | Australia | Inpatient | Cross-Sectional | 876 | 42 | Median:86; Range:76-89 | 9 |
| Feng et al. (2017) | USA | - | Cross-Sectional | 37570 | 58.4 | Range:18-64 | 10 |
| Hung et al. (2017) | Taiwan | ≥ 1 setting | Cohort | 395 | 24 | Mean (SD): 87 (6) | 5 |
| McLean et al. (2017) | UK | Outpatient | Case-Control | 510502 | 54.1 | Minimum:55 | 7 |
| Mizokami et al. (2017) | Japan | Inpatient | Cross-Sectional | 45 | 5667 | Mean (SD): 78 (7) | 9 |
| Park et al. (2017) | South Korea | Community | Case-Control | 11124 | 71.8 | Minimum:65 | 8 |
| Park et al. (2017) | South Korea | - | Case-Control | 31045 | 80.2 | Mean: 78 | 8 |
| Wawruch et al. (2017) | Slovakia | Inpatient | Cross-Sectional | 854 | 64.6 | Mean (SD): 76 (7); Min: 65 | 9 |
| Yeh et al. (2017) | Taiwan | - | Cross-Sectional | 318 | 100 | Mean (SD): 30 (4) | 8 |
| Yu et al. (2017) | Taiwan | Inpatient | Case-Control | 10883 | 60.5 | Mean: 78 | 8 |
| Asranna et al. (2018) | India | - | Cohort | 1547 | 100 | Mean (SD): 26 (4.4) | 5 |
| Baek & Shin (2018) | South Korea | Outpatient | Cross-Sectional | 746980 | - | All age groups | 10 |
| Chiapella et al. (2018) | Argentina | Community | Cross-Sectional | 3972 | - | Minimum:65 | 8 |
| Cho et al. (2018) | South Korea | - | Cross-Sectional | 1122080 | 58.92 | Minimum:65 | 10 |
| Faught et al. (2018) | USA | - | Cohort | 29226 | 51.1 | Mean (SD): 36 (27) | 7 |
| Fontanella et al. (2018) | USA | ≥ 1 setting | Cross-Sectional | 25062 | - | Mean (SD): 46 (12) | 10 |
| Guilcher et al. (2018) | Canada | Inpatient | Cross-Sectional | 418 | 37 | Mean (SD): 75 (6) | 10 |
| Kadra et al. (2018) | UK | - | Cross-Sectional | 6857 | 41.1 | Minimum:16 | 6 |
| Kadra et al. (2018) | UK | - | Cohort | 5523 | 53.4 | Mean (SD): 41 (15) | 10 |
| McIsaac et al. (2018) | Canada | Inpatient | Cohort | 266499 | 53 | Minimum:66 | 7 |
| Morin et al. (2018) | Sweden | ≥ 1 setting | Cross-Sectional | 1742336 | 55.1 | Mean (SD): 75 (8) | 9 |
| Subesinghe et al. (2018) | UK | - | Cross-Sectional | 6460 | 73.6 | Mean (SD): 57 (13) | 9 |
| Wastesson et al. (2018) | Sweden | ≥ 1 setting | Cross-Sectional | 822619 | 58.9 | Minimum:75 | 9 |
| Ivanova et al. (2019) | Sweden | Community | Cross-Sectional | 315120 | 54.7 | Mean: 74; Range: 65-114 | 10 |
| Thunander & Hedborg (2019) | Sweden | - | Cross-Sectional | 5289 | 57.4 | Range:18-84 | 9 |
| van den Akker et al. (2019) | Belgium | - | Cross-Sectional | 152270 | 50 | Minimum:0 | 9 |
| Wastesson et al. (2019) | Sweden | - | Cross-Sectional | 1752022 | 59.1 | Mean (SD): 77 (8) | 10 |
| Constantine et al. (2010) | USA | - | Cross-Sectional | 51756 | 59.5 | Mean:42 | 9 |

*Italy, France, South America, Spain, Sweden. † India, Malaysia, and Thailand, China, Hong Kong, Japan, Korea, Singapore, Taiwan. UK: United Kingdom; US: United States of America. Empty cells indicate that sufficient information was not provided in primary study.
