# Supplementary material for: Prevalence and factors associated with polypharmacy: a systematic review and Meta-analysis
Source: BMC Geriatr. 2022 Jul 19;22:601. doi: 10.1186/s12877-022-03279-x (PMC9297624; doi:10.1186/s12877-022-03279-x)
Supplement: Supplementary file 3 — Additional File 3. Descriptive Definitions and Measurement Indicators of Polypharmacy and Alternative Terms across Studies. [file 12877_2022_3279_MOESM3_ESM.docx]

## Additional File 3. Descriptive Definitions and Measurement Indicators of Polypharmacy and Alternative Terms across Studies

| **Authors** | **Terminology used** | **Descriptive definition** | **Measurement indicator** | **Sub-indicator definition** | **Type of drug assessment** | **Name of the drug** |
| --- | --- | --- | --- | --- | --- | --- |
| Källén et al. (1989) | Polytherapy | ≥ 3 antiepileptic medications used in combination during the 1^st^ trimester of pregnancy | Simultaneous | Concurrent use of medications with at least 1day overlap | Intra-class | Antiepileptics |
| Bjerrum et al. (1997) | Minor polypharmacy | 2 to 4 medications used concurrently | Simultaneous | Concurrent use of medications with at least 1day overlap | Any medication | - |
|  | Major polypharmacy | ≥ 5 medications used concurrently |  |  |  |  |
| Bjerrum et al. (1998) | Minor polypharmacy | 2 to 4 medications used concurrently | Simultaneous | Concurrent use of medications with at least 1day overlap | Both inter and intra-class | ATC 2^nd^ and 5^th^ levels |
|  | Major polypharmacy | ≥ 5 medications used concurrently |  |  |  |  |
| Veehof et al. (2000) | Polypharmacy | ≥ 2 medications simultaneously used for ≥ 60 days per quarter per year | Simultaneous | Concurrent use of medications with at least 240 days overlap | Unspecified | - |
|  | Minor polypharmacy | 2 to 3 medications simultaneously used for ≥ 60 days per quarter per year |  |  |  |  |
|  | Moderate polypharmacy | 4 to 5 medications simultaneously used for ≥ 60 days per quarter per year |  |  |  |  |
|  | Major polypharmacy | ≥ 6 medications simultaneously used for ≥ 60 days per quarter per year |  |  |  |  |
|  | Long term polypharmacy | ≥ 2 medications simultaneously used with overlap of ≥ 60 days per quarter per year (a total of ≥ 240 days a year) |  |  |  |  |
| Weissman (2002) | Polypharmacy | ≥ 2 antipsychotics drugs used concomitantly | Simultaneous | Concurrent use of medications with at least 1day overlap | Intra-class | Antipsychotics |
| Hamann et al. (2003) | Comedication | ≥ 1 different medication prescribed in addition to an antipsychotic within a time interval of 45 days before or following the antipsychotic prescription | Cumulative | Sum of different medications taken over 14 days | Both inter and intra-class | Antipsychotics: amisulpride, clozapine, olanzapine, quetiapine, risperidone, zotepine and typical antipsychotics any other class e.g. cardiovascular |
|  | Polypharmacy | ≥ 2 antipsychotics within a time interval of 14 days |  |  |  |  |
| Jaffe & Levine (2003) | Co-prescribing | ≥ 2 medications with overlap for at least 28 days | Simultaneous | Concurrent use of medications with at least 28 days overlap | Intra-class | Antipsychotics |
| De las Cuevas & Sanz (2004) | Polypharmacy | ≥ 2 psychiatric medication class used concurrently | Simultaneous | Concurrent use of medications with at least 1day overlap | Both inter and intra-class | Psychotropics: antipsychotics, antidepressants and tranquillisers: including hypnotics, benzodiazepines |
|  | Same-class polypharmacy | ≥ 2 medications from the same medication class |  |  |  |  |
|  | Multi-class polypharmacy | ≥ 2 medications used at their full therapeutic doses from different medication classes for the same symptom cluster |  |  |  |  |
|  | Adjunctive polypharmacy | ≥ 2 medications with one used to treat the side effects or secondary symptoms of another medication from a different medication class |  |  |  |  |
|  | Augmentation polypharmacy | ≥ 2 medications with one used at a lower-than-normal dose along with another medication from a different medication class at its full therapeutic dose, for the same symptom cluster or the addition of a medication that would not be used alone for the same symptom cluster |  |  |  |  |
|  | Total polypharmacy | The total count of medications or total drug load |  |  |  |  |
| Dolk et al. (2008) | Polytherapy | ≥ 2 medications in the first trimester of pregnancy | Cumulative | Sum of different medications taken over 90 days | Intra-class | Antiepileptics |
| Malm et al. (2004) | Polypharmacy | - | Cumulative | Sum of different medications taken over 280 days | Inter-class | ATC 3rd level |
| French et al. (2005) | Polypharmacy | ≥ 2 medication classes of cardiovascular, CNS, musculoskeletal | Simultaneous | Concurrent use of medications with at least 1day overlap | Inter-class | CNS, cardiovascular, musculoskeletal system |
| Preskorn et al. (2005) | Multiple medication use | ≥ 2 concurrent medications when the number of days of supply equals or exceed the number of days since the last refill | Simultaneous | Concurrent use of medications with at least 1day overlap | Any medication | drugs that could interact systemically or gastrointestinally e.g. acetaminophen and codeine in a combination |
| Åstrand et al. (2006) | Polypharmacy | - | Cumulative | Sum of different medications taken over 450 days | Any medication | ATC 5th level |
| Åstrand et al. (2007) | Polypharmacy | - | Cumulative | Sum of different medications taken over 450 days | Any medication | ATC 5th level |
| Targownik et al. (2007) | Polypharmacy | ≥ 10 concomitant medications | Simultaneous | Concurrent use of medications with at least 45 days overlap | Any medication | nonsteroidal anti-inflammatory drugs (NSAIDs), acetylsalicylic acid (ASA), clopidrogel, warfarin, antispasmodic/promotility agents, and selective serotonin reuptake inhibitors (SSRIs) |
| Carey et al. (2008) | Polypharmacy | ≥ 2 medications, each with ≥ 3 prescriptions in a year | Cumulative | Sum of different medications taken over 360 days | Unspecified | - |
| Gidal et al. (2009) | Concomitant, polytherapy, polypharmacy, Adjunctive polypharmacy | ≥ 2 concomitant medication | Simultaneous | Concurrent use of medications with at least 1day overlap | Inter-class | Non epileptic drugs with either enzyme-inducing AEDs (EIAEDs) :phenytoin, carbamazepine, phenobarbital, and primidone, OR non– enzyme-inducing AEDs (NEIAEDs): valproate, tiagabine, levetiracetam, lamotrigine, gabapentin, topiramate, oxcarbazepine, and zonisamide |
| Haider et al. (2009) | Polypharmacy | 5 to 9 concurrent medications | Simultaneous | Concurrent use of medications with at least 1day overlap | Any medication |  |
|  | Excessive polypharmacy | ≥ 10 concurrent medications |  |  |  |  |
| Hsieh & Huang (2009) | Polytherapy | ≥ 2 antileptic medications as concomitant therapy | Simultaneous | Concurrent use of medications with at least 1day overlap | Intra-class | Antiepileptics |
| Constantine et al. (2010) | Polypharmacy | ≥ 2 antipsychotics simultaneously used >60 days with no gaps >15 days | Simultaneous | Concurrent use of medications with at least 60 days overlaps and allowed gap of 15 days | Intra-class | Antipsychotics |
| Hovstadius et al. (2010) | Polypharmacy | ≥ 5 medications during a 3-month period | Cumulative | Sum of different medications taken over 120 days | Any medication | - |
|  | Excessive polypharmacy | ≥ 10 medications during a 3-month period |  |  |  |  |
| Lai et al. (2010) | Polypharmacy | ≥ 5 medications used on daily average calculated over 2 years | Cumulative | Sum of different medications taken over 730 days | Any medication | - |
| Moisan & Grégoire (2010) | Polytherapy | ≥ 2 atypical antipsychotic, or 1 atypical and 1 typical antipsychotic | Simultaneous | Concurrent use of medications with at least 1day overlap | Intra-class | Antipsychotics |
| Slabaugh et al. (2010) | Polypharmacy | Overlapping treatment with ≥ 5 medications occurring for at least 1 day | Simultaneous | Concurrent use of medications with at least 1day overlap | Any medication | ATC 5^th^ level |
| Charlton et al. (2011) | Polytherapy | ≥ 2 antileptic medications | Simultaneous | Concurrent use of medications with at least 1day overlap | Intra-class | Antiepileptic |
| Hoffmann et al. (2011) | Polypharmacy | - | Cumulative | Sum of different medications taken over 120 days | Intra-class | Antidementia drugs |
| Kragh et al. (2011) | Polypharmacy | ≥ 5 medications | Cumulative | Sum of different medications taken over 180 days | Intra-class | Psychotropics: sedative and hypnotic, antidepressive, antipsychotic, benzodiazepine (all and long-acting)), cardiovascular (excluding lipid-lowering drugs), anticholinergic, antiepileptic, antiparkinson, and opioids |
|  | Excessive polypharmacy | ≥ 10 medications |  |  |  |  |
| Kulaga et al. (2011) | Polytherapy | ≥ 2 antileptic medications in one prescription filled in the 1^st^ trimester | Simultaneous | Concurrent use of medications with at least 1day overlap | Intra-class | antiepileptic: carbamazepine, clobazam, clonazepam, ethosuximide, gabapentin, lamotrigine, phenobarbital, phenytoin, primidone, topiramate ,valproic acid or vigabatrin |
| Lai et al. (2011) | Polypharmacy | ≥ 5 medications used on daily average calculated over 2 years | Cumulative | Sum of different medications taken over 730 days | Any medication | - |
| Landmark et al. (2011) | Polytherapy | ≥ 2 concomitant medications within one specific month | Simultaneous | Concurrent use of medications with at least 1day overlap | Intra-class | Antiepileptics |
| Pergolizzi Jr et al. (2011) | Concurrent medication | ≥ 2 concurrent opioids metabolized by the CYP450 system (codeine, | Simultaneous | Concurrent use of medications with at least 1day overlap | Intra-class | - |
|  |  | fentanyl, hydrocodone, methadone, oxycodone, or tramadol) |  |  |  |  |
|  |  | as concomitant therapy with at least a one day |  |  |  |  |
|  |  | overlap in days supplied within the 30-day window  of time |  |  |  |  |
| Sanglier et al. (2011) | Polypharmacy | ≥ 4 different, non antidepressant compounds during a month | Cumulative | Sum of different medications taken over 30 days | Any medication: | Excluding antidepressants |
| Andrew et al. (2012) | Polytherapy | ≥ 2 antileptic medications | Simultaneous | Concurrent use of medications with at least 1day overlap | Intra-class | Antiepileptics |
| Baandrup et al. (2012) | Polypharmacy | ≥ 2 antipsychotics drugs used concomitantly | Simultaneous | Concurrent use of medications with at least 1day overlap | Intra-class | Antiepileptics |
| Lai et al. (2012) | Polypharmacy | ≥ 5 medications used on daily average calculated over 2 years | Cumulative | Sum of different medications taken over 730 days | Any medication | - |
| Tiihonen et al. (2012) | Polypharmacy | ≥ 2 antipsychotics drugs used concomitantly | Simultaneous | Concurrent use of medications with at least 1day overlap | Both inter and intra-class | antipsychotics, antidepressants, benzodiazepines |
| Xiang et al. (2012) | Polypharmacy | ≥ 2 antipsychotics drugs used concomitantly | Simultaneous | Concurrent use of medications with at least 1day overlap | Intra-class | Antipsychotics |
| Blozik et al. (2013) | Polypharmacy | ≥ 5 medications reimbursed in a quarter of a year | Cumulative | Sum of different medications taken over 120 days | Unspecified | - |
| Calderón-Larrañaga (2013) | Polypharmacy | ≥ 3 concurrent medications at 2 different months | Simultaneous | Concurrent use of medications with at least 1day overlap | Intra-class | ATC 3^rd^ level:  CNS (depression-anxiety,), cardiovascular, acute respiratory infection (ARI), chronic obstructive pulmonary disease (COPD), rhinitis-asthma, pain, and menopause |
| Curkendall et al. (2013) | Polytherapy | ≥ 1 antidiabetic medications prescribed in 90 days after an index drug | Cumulative | Sum of different medications taken over 90 days | Intra-class | Antidiabetics |
| Franchi et al. (2013) | Chronic polypharmacy | ≥ 5 medications in 1 month for at least 6 months (consecutive or not) in a year | Simultaneous | Concurrent use of medications with at least 180 days overlap | Inter-class | ATC 4^th^ level |
| Gören et al. (2013) | Polypharmacy | ≥ 2 medications concomitantly used with ≥ 90 days overlap | Simultaneous | Concurrent use of medications with at least 90 days overlap | Intra-class | Antipsychotics |
| Lizano-Díez et al. (2013) | Poly medicate | ≥ 16 medications as active principles in a month | Cumulative | Sum of different medications taken over 30 days | Inter-class | - |
| Onishi et al. (2013) | Polypharmacy | ≥ 2 combinations of 4 types of medications from multiple classes used for treating major depression | Cumulative | Sum of different medications taken over 360 days | Inter-class | first- and/or second-generation antidepressants; benzodiazepine; sulpiride and antipsychotics |
| Palmsten et al. (2013) | Polytherapy | ≥ 2 medication classes exposed concomitantly or sequentially with an SSRI and another class or non-SSRIs | Cumulative | Sum of different medications taken over 90 days | Intra-class | antidepressants |
| Suokas et al. (2013) | Polypharmacy | ≥ 2 antipsychotics for over 60 days | Simultaneous | Concurrent use of medications with at least 1day overlap | Intra-class | Antipsychotics |
| Wong et al. (2013) | Polytherapy | ≥ 2 antihypertensive medications | Simultaneous | Concurrent use of medications with at least 1day overlap | Intra-class | [alpha-blockers, beta-blockers, thiazide diuretics, calcium channel blockers (CCBs), angiotensin-converting enzyme inhibitors (ACEIs), angiotensin receptor blockers (ARBs)] |
| Degli Esposti et al. (2014) | Polytherapy | ≥ 2 antipsychotic medications used simultaneously for 1 year either as switch therapy or add-on treatment | Cumulative | Sum of different medications taken over 360 days | Intra-class | Antipsychotics |
| Fano et al. (2014) | Minor polypharmacy | 2 to 3 medications, each used for ≥ 60 days of therapy in a year | Cumulative | Sum of different medications taken over 60 days | Inter-class |  |
|  | Moderate polypharmacy | 4 to 5 medications each with at least 60 days of therapy in a year |  |  |  |  |
|  | Major polypharmacy | ≥ 6 medications each with at least 60 days of therapy in a year |  |  |  |  |
| Fereshtehnejad et al. (2014) | Polypharmacy | ≥ 5 medications | Simultaneous | Concurrent use of medications with at least 1day overlap | Inter-class | Cholinesterase inhibitors, NMDA antagonists, cardiovascular drugs, antidepressants, antipsychotics, anxiolytics and hypnotics |
| Franchi et al. (2014) | Polypharmacy | ≥ 5 medications from different active substances | Cumulative |  |  |  |
|  | Chronic polypharmacy | ≥ 5 medications from different active substances with ≥ 4 packages of each drug substance |  |  |  |  |
| Frandsen et al. (2014) | Polytherapy | ≥ 2 medications, each with ≥ 3 prescriptions during the study period | Simultaneous | Concurrent use of medications with at least 1day overlap | Both inter and intra-class | Selective serotonin re-uptake inhibitors (SSRIs) (ATC-code: N06AB), serotonin-noradrenaline re-uptake inhibitors (SNRIs) (06AX), tricyclic antidepressants (TCAs) (N06AA), benzodiazepines (BZDs) (N05BA, N03AE01,N05CD), benzodiazepine-like drugs (BZD-like) (N05CF),first-generation antipsychotics (FGAs) (N05A, excluding N05AX08-12-13, N05AL05, N05AH02-03-04-05,N05AE03e04, N05AN01) and second-generation antipsychotics (SGAs) (N05AX08-12-13, N05AL05, N05AH02-03-04-05, N05AE03-04) |
| Gamble et al. (2014) | Polypharmacy | ≥ 5 medications assessed at baseline and 90-day intervals of a year | Simultaneous | Concurrent use of medications with at least 1day overlap | Any medication |  |
| Guidoni et al. (2014) | Polytherapy | ≥ 5 concomitant medications | Simultaneous | Concurrent use of medications with at least 1day overlap | Any medication | ATC 1^st^ level: warfarin and other drugs |
| Helgadóttir et al. (2014) | Polypharmacy | ≥ 5 medications | Cumulative | Sum of different medications taken over 90 days | Inter-class | - |
| Hovstadius et al. (2014) | Polypharmacy | ≥ 3 concomitant psychotropic drugs used regularly or as needed | Simultaneous | Concurrent use of medications with at least 1day overlap | Both inter and intra-class | - |
|  | Excessive polypharmacy | ≥ 10 concomitant psychotropic drugs used regularly or as needed |  |  |  |  |
| Kim et al. (2014) | Polypharmacy | ≥ 6 concurrent/ simultaneous medications | Simultaneous | Concurrent use of medications with at least 1day overlap | Any medication | - |
|  | Major polypharmacy | ≥ 11 concurrent medications |  |  |  |  |
|  | Excessive polypharmacy | ≥ 21 concurrent medications |  |  |  |  |
| Monégat et al. (2014) | Polypharmacy | ≥ 5 medications prescribed simultaneously (one day at random, an average day of year, an average day over 20 days) or cumulatively (over the quarter average. over 4 quarters) or continuously (≥3 times a year) | Simultaneous | Concurrent use of medications with at least 1day overlap, on a random or an average day per year or 20 days | Any medication | ATC 5^th^ level |
|  |  |  | Cumulative | Sum of different medications taken over 120 days |  |  |
|  |  |  | Continuous | Sum of different medications taken in a 120-day period and at least 3 times a year with a total of 360 days |  |  |
| Onder et al. (2014) | Polypharmacy | 5 to 9 medications at ATC 2^nd^ level or ≥10 medications at ATC 5^th^ level over a quarter of a year | Cumulative | Sum of different medications taken over 120 days | Any medication | ATC 5^th^ level |
| Pottegård et al. (2014) | Polypharmacy | ≥ 8 medications | Cumulative | Sum of different medications taken over 360 days | Any medication | - |
| Rossini et al. (2014) | Polypharmacy | ≥ 10 medications | Cumulative | Sum of different medications taken over 360 days | Both inter and intra-class | loop diuretics, NSAIDs, other analgesics [paracetamol (acetaminophen) or opioids], oral antidiabetics, insulin, statins (HMG-CoA reductase inhibitors), antidepressants, b-blockers, calcium channel antagonists, ACE inhibitors, angiotensin receptor blockers (sartanics), other antihypertensive medications, gastroprotection’s, thyroid hormones, antiparkinson or antiepileptic drugs, drugs for chronic obstructive pulmonary disease (COPD), systemic corticosteroids or inhaled corticosteroids |
| Wang et al. (2014) | Polypharmacy |  | Simultaneous | Concurrent use of medications with at least 1day overlap | Both inter  and intra-class | Benzodiazepines, z hypnotic agents: alprazolam, bromazepam, brotizolam, chlordiazepoxide, clobazam, clonazepam, clorazepate, cloxazolam,  diazepam, estazolam, fludiazepam, flunitrazepam, flurazepam, lorazepam, lormetazepam, medazepam, midazolam,  nimetazepam, nitrazepam, nordazepam, oxazepam, oxazolam, prazepam, triazolam, zaleplon, zolpidem, and zopiclone |
| Gaviria et al. (2015) | Long term polypharmacy | ≥ 2 antipsychotics simultaneously used with overlap of >120 days | Simultaneous | Concurrent use of medications with at least 90 days overlap | Intra-class | Antipsychotics |
| Laflamme et al. (2015) | Polypharmacy | ≥ 5 medications | Cumulative | Sum of different medications taken over 30 days | Both inter and intra-class | FRIDS: vasodilators used in cardiac diseases, antihypertensive drugs, diuretics, beta blocking agents, calcium channel blockers, agents acting on the renin-angiotensin system, alpha adrenoreceptor antagonists (for benign prostatic hypertrophy), opioids, dopaminergic agents (anti-Parkinson drugs), antipsychotics excl. lithium, anxiolytics, hypnotics and sedatives and antidepressants |
| Lin et al. (2015) | Polypharmacy | - | Cumulative | Sum of different medications taken over an unspecified period | Intra-class | Psychotropics |
| Lu et al. (2015) | Polypharmacy | ≥ 5 concomitant medications with at least 28 days of supply | Simultaneous | Concurrent use of medications with at least 1day overlap | Any medication | - |
|  | Excessive polypharmacy | ≥ 10 concomitant medication with at least 28 days of supply |  |  |  |  |
| Salahudeen et al. (2015) | Polypharmacy | ≥ 5 concurrent medications dispensed for ≥ 365 days | Simultaneous | Concurrent use of medications with at least 365 days overlap | Intra-class | anticholinergics |
| van de Vorst et al. (2015) | Polypharmacy | ≥ 5 regular medications (excluding temporary drugs or antibiotics) | Simultaneous | Concurrent use of medications with at least 1day overlap | Any medication | - |
| Baandrup et al. (2016) | Polypharmacy | ≥ 2 antipsychotics compounds collected within 2 successive intervals of 60 days | Cumulative | Sum of different medications taken over 60 days | Intra-class | Antiepileptics |
| Chang et al. (2016) | Combination therapy, polytherapy | ≥ 2 concomitant psychotropic medication from same class for > 6 weeks during a 1-year period | Simultaneous | Concurrent use of medications with at least 42 days overlap | Both inter and intra-class | Antipsychotics, antidepressants, mood stabilizers, benzodiazepines |
| Ekstam & Elmståhl (2016) | Polypharmacy | ≥ 5 medications | Simultaneous | Concurrent use of medications with at least 1day overlap | Intra-class | Psychotropic (including sedative/hypnotic, ant depressive, antipsychotic [excluding lithium], and benzodiazepine), cardiovascular (excluding lipid-lowering drugs), anticholinergic, antiepileptic, antiparkinsonian, and opioids |
|  | Major polypharmacy | ≥ 10 medications |  |  |  |  |
| Horváth et al. (2016) | Polytherapy | ≥ 3 antiepileptic or CNS medications | Simultaneous | Concurrent use of medications with at least 1day overlap | Intra-class | Antiepileptics |
| Sinnige et al. (2016) | Polypharmacy | ≥ 5 medications, each with ≥ 4 prescriptions or prescribed for ≥ 90 days, used simultaneously ≥ 1 day in a year | Simultaneous | Concurrent use of medications with at least 1day overlap | Any medication: | ATC 3^rd^ level: Excluding topicals antibiotics |
| van den Bemt et al. (2016) | Polypharmacy | ≥ 5 concomitant medications | Simultaneous | Concurrent use of medications with at least 1day overlap | Unspecified |  |
| van Erning et al. (2016) | Polypharmacy | - | Cumulative | Sum of different medications taken over 120 days | Intra-class | ATC 2^nd^ level: anticonstipations, antithrombotic, anti acids, antibacterial, renin–angiotensin system (RAS)-related agents, beta-blockers, lipid-modifying agents, diuretics, psycholeptics, antianaemics. excluding non-therapeutic products |
| Abe et al. (2017) | Polypharmacy | ≥ 5 medications with renal or hepatic adverse effects | Cumulative | Sum of different medications taken over 360 days | Any medication | ATC 1^st^ level: |
|  |  |  |  |  |  | Hepatic and renal drugs |
| Broeks et al. (2017) | Polypharmacy | ≥ 2 medications concomitantly used with ≥ 90 days overlap | Simultaneous | Concurrent use of medications with at least 90 days overlaps and a grace period of 28 days | Inter-class | Psychotropics |
| Byrne (2017) | Co-prescribing | ≥ 3 chronic medications dispensed in a year for each drug class (second-level ATC) | Cumulative | Sum of different medications taken over 360 days | Inter-class | ATC 2^nd^ level |
| Caughey et al. (2017) | Hyper polypharmacy | ≥ 10 unique medicines dispensed in the 120 days before and after hospitalization | Cumulative | Sum of different medications taken over 120 days | Intra-class | Antidiabetic, cardiovascular |
| Feng et al. (2017) | Polypharmacy | ≥ 5 concurrent medications from different drug classes per day for ≥60 days in 72 consecutive days | Simultaneous | Concurrent use of medications with at least 60 days overlaps and allowed gap of 12 days | Inter-class |  |
| Hung et al. (2017) | Polypharmacy | ≥ 5 concomitant medications | Cumulative | Sum of different medications taken over 360 days | Any medication | - |
| McLean et al. (2017) | Polypharmacy | ≥ 5 medications authorised for repeat prescription and issued within 84 days | Cumulative | Sum of different medications taken over 84 days | Any medication | - |
| Mizokami et al. (2017) | Polypharmacy | ≥ 5 medications at discharge | Simultaneous | Concurrent use of medications with at least 1day overlap | Intra-class | ATC 2^nd^ and 3^rd^ levels |
| Park et al. (2017) | Polypharmacy | ≥ 5 medications used on a daily average calculated over 1 year | Cumulative | Sum of different medications taken over 720 days | Intra-class | Antihypertension and antidiabetic |
| Park et al. (2017) | Polypharmacy | ≥ 5 medications used on a daily average calculated over 1 year | Cumulative | Sum of different medications taken over 360 days | Any medication | - |
| Wawruch et al. (2017) | Polypharmacy | ≥ 6 concurrent/ simultaneous medications | Simultaneous | Concurrent use of medications with at least 1day overlap | Any medication | - |
| Yeh et al. (2017) | Polytherapy | ≥ 2 antileptic medications | Simultaneous | Concurrent use of medications with at least 1day overlap | Intra-class | Antiepileptics |
| Yu et al. (2017) | Polypharmacy | ≥ 2 benzodiazepines or any benzodiazepine plus non-benzodiazepine hypnotics used within 30 days | Cumulative | Sum of different medications taken over 30 days | Both inter and intra-class | Benzodiazepines and non-benzodiazepine hypnotics |
| Asranna et al. (2018) | Polytherapy | ≥ 2 antileptic medications | Simultaneous | Concurrent use of medications with at least 1day overlap | Intra-class | Antiepileptics |
| Baek & Shin (2018) | Polypharmacy | ≥ 6 concurrent/ simultaneous medications | Simultaneous | Concurrent use of medications with at least 1day overlap | Any medication | - |
| Chiapella et al. (2018) | Minor polypharmacy | 2 to 4 medications used simultaneously (on a random day within 6 months) or cumulatively (monthly average in ≥ 3 consecutive months) or continuously (≥ 2 continues period of 3 months) | Simultaneous  Cumulative  Continues | Concurrent use of medications with at least 1day overlap  Sum of different medications taken over 90 days  Sum of different medications taken in a 90-day period and at least 2 times a year with a total of 180days | Any medication | ATC 5th level |
|  | Major polypharmacy | ≥ 5 medications used simultaneously (on a random day within 6 months) or cumulatively (monthly average in ≥ 3 consecutive months) or continuously (≥ 2 continues period of 3 months) |  |  |  |  |
| Cho et al. (2018) | Polypharmacy | prescription having 5-9 active ingredients | Cumulative | Sum of different medications taken over 360 days | Inter-class | Psychotropics |
|  | Excessive polypharmacy | prescription with ≥ 10 active ingredients |  |  |  |  |
| Faught et al. (2018) | Polytherapy | ≥ 2 antileptic medications used simultaneously ≥ 90 days | Simultaneous | Concurrent use of medications with at least 90 days overlap | Intra-class | antiepileptics |
| Fontanella et al. (2018) | Polypharmacy | ≥ 2 antipsychotics simultaneously used with overlap of >90 days at anytime during the study period and an allowable 14-day gap between prescription fills | Simultaneous | Concurrent use of medications with at least 90 days overlaps and allowed gap of 14 days | Intra-class | Antipsychotics |
|  | Long term polypharmacy | ≥ 2 antipsychotics simultaneously used with overlap of >90 days at anytime during the study period and an allowable 14-day gap between prescription fills |  |  |  |  |
| Guilcher et al. (2018) | Polypharmacy | ≥ 10 medications | Cumulative | Sum of different medications taken over 360 days | Inter-class |  |
| Kadra et al. (2018) | Long term polypharmacy | ≥ 2 antipsychotic agents concomitantly prescribed for ≥ 6 months | Simultaneous | Concurrent use of medications with at least 180 days overlap | Intra-class | Antipsychotics |
| Kadra et al. (2018) | Polypharmacy | ≥ 2 antipsychotics drugs taken during the inpatient stay and in the 6 weeks following discharge | Cumulative | Sum of different medications taken over 42 days | Intra-class | Antipsychotics |
| McIsaac et al. (2018) | Polypharmacy | - | Cumulative | Sum of different medications taken over 90 days | Any medication | - |
| Morin et al. (2018) | Polypharmacy | ≥ 5 concurrent medications | Simultaneous | Concurrent use of medications with at least 1day overlap | Inter-class | - |
|  | Excessive polypharmacy | ≥ 10 concurrent medications |  |  |  |  |
| Subesinghe et al. (2018) | Polypharmacy | - | Simultaneous | Concurrent use of medications with at least 1day overlap | Unspecified | - |
| Wastesson et al. (2018) | Polypharmacy | ≥ 5 medications | Simultaneous | Concurrent use of medications with at least 1day overlap | Both inter and intra-class | ATC 5th level |
|  | Excessive polypharmacy | ≥ 10 medications |  |  |  |  |
| Ivanova et al. (2019) | Polypharmacy | ≥ 5 chronic medications concurrently dispensed ≥ 2 times during a 6‐month period | Simultaneous | Concurrent use of medications with at least 1day overlap | Intra-class | ATC 1^st^ level: group c: cardiovascular |
|  | Excessive polypharmacy | ≥10 chronic medications concurrently dispensed ≥ 2 times during a 6‐month period |  |  |  |  |
| Thunander & Hedborg (2019) | Polypharmacy | ≥ 5 medications including antidepressants | Cumulative | Sum of different medications taken over 90 days | Inter-class | ATC A01-16, B01-06, C01-C10, D01-D10, G01-04, H01-05, J01-07, L01-04, M01-M09, N01-02, N03-05, N07, R01-07, S01-03, diabetes (A10), and in main group N, analgesics (N01-N02) and antidepressants (N06A). The ATC groups P (Antiparasitic products, foremost preventive and over-the-counter (OTC) drugs) and V (Various, foremost diagnostic aids and antidotes) |
| van den Akker et al. (2019) | Polypharmacy | ≥ 5 medications in a year | Cumulative | Sum of different medications taken over 360 days | Inter-class | ATC 4^th^ level |
| Wastesson et al. (2019) | Polypharmacy | ≥ 5 medications in a month | Cumulative | Sum of different medications taken over 30 days | Any medication | ATC 5th level |

\ATC:  Anatomical Therapeutic Chemical
