# Supplementary figures and images for: Prevalence and factors associated with polypharmacy: a systematic review and Meta-analysis
Source: BMC Geriatr. 2022 Jul 19;22:601. doi: 10.1186/s12877-022-03279-x (PMC9297624; doi:10.1186/s12877-022-03279-x)

## Additional file 4. Prevalence of Polypharmacy Associated with Different Age Subgroups


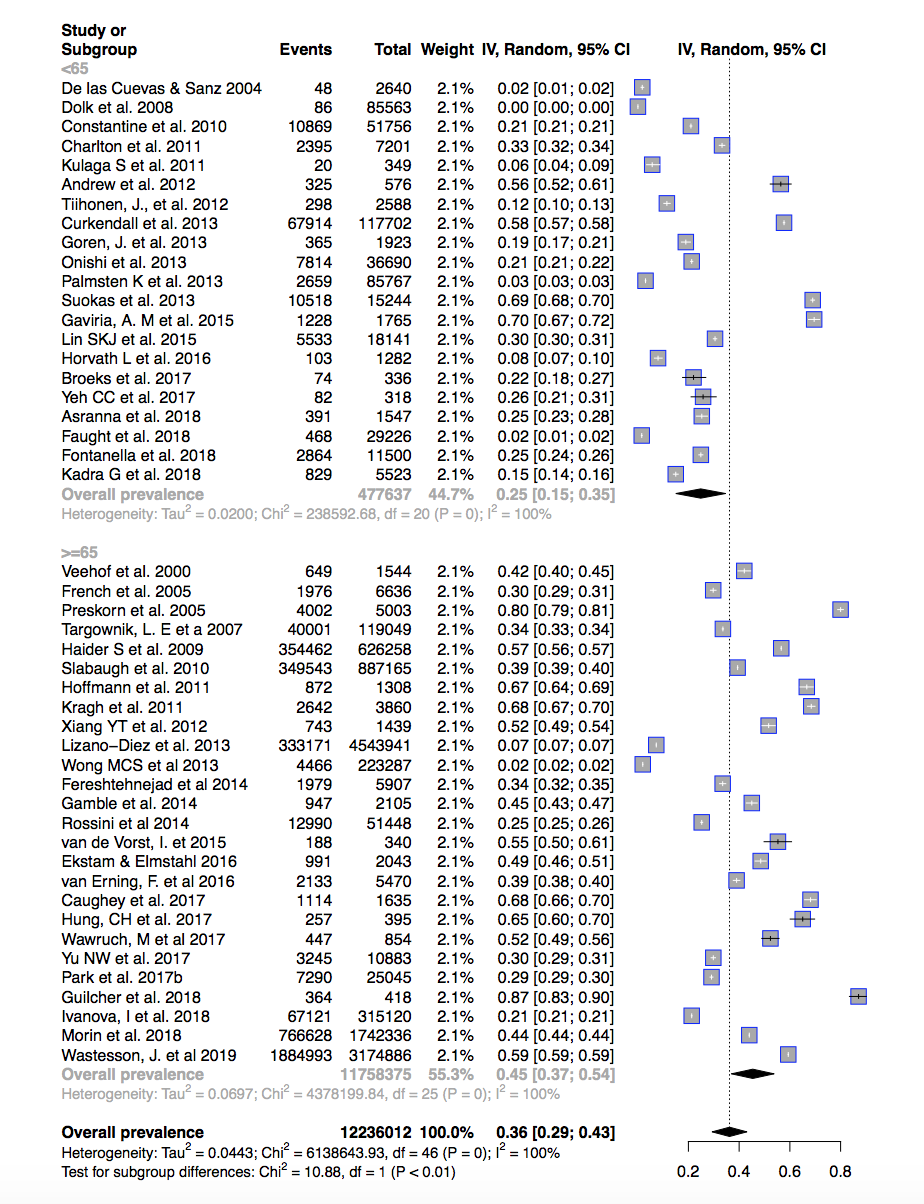

Supplement: Supplementary file 4 — Additional file 4. Prevalence of Polypharmacy Associated with Different Age Subgroups. [file 12877_2022_3279_MOESM4_ESM.docx]

## Additional file 6. Prevalence of Polypharmacy Associated with Settings of Study

##
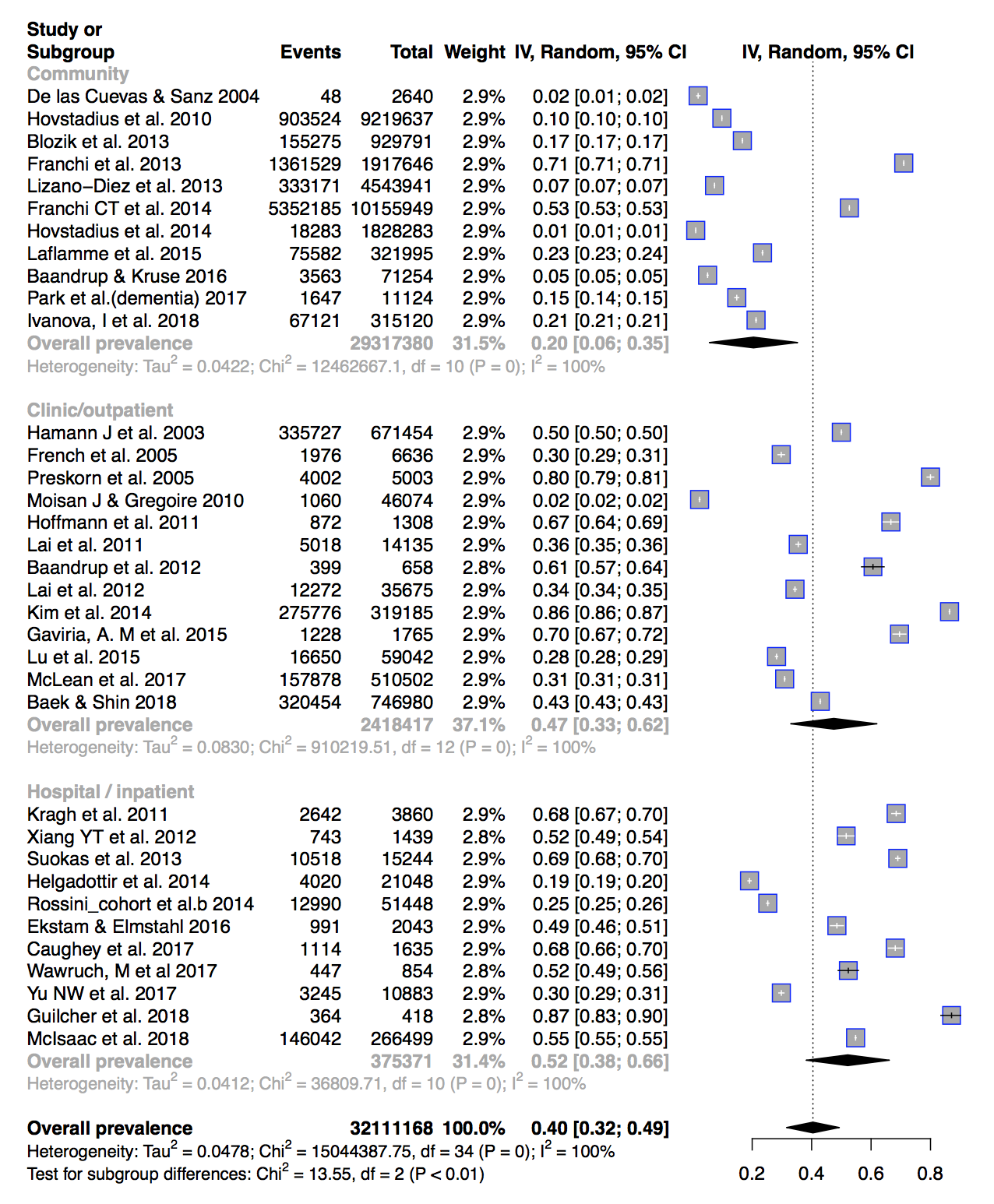

Supplement: Supplementary file 6 — Additional file 6. Prevalence of Polypharmacy Associated with Settings of Study. [file 12877_2022_3279_MOESM6_ESM.docx]

## Additional file 7. Prevalence of Polypharmacy Associated with Sex

##
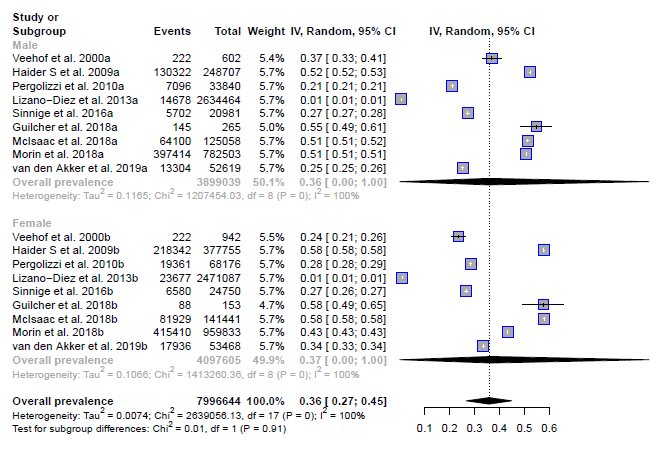

Supplement: Supplementary file 7 — Additional file 7. Prevalence of Polypharmacy Associated with Sex. [file 12877_2022_3279_MOESM7_ESM.docx]

## Additional file 8. Prevalence of Polypharmacy Associated with Study Geographical Location

##
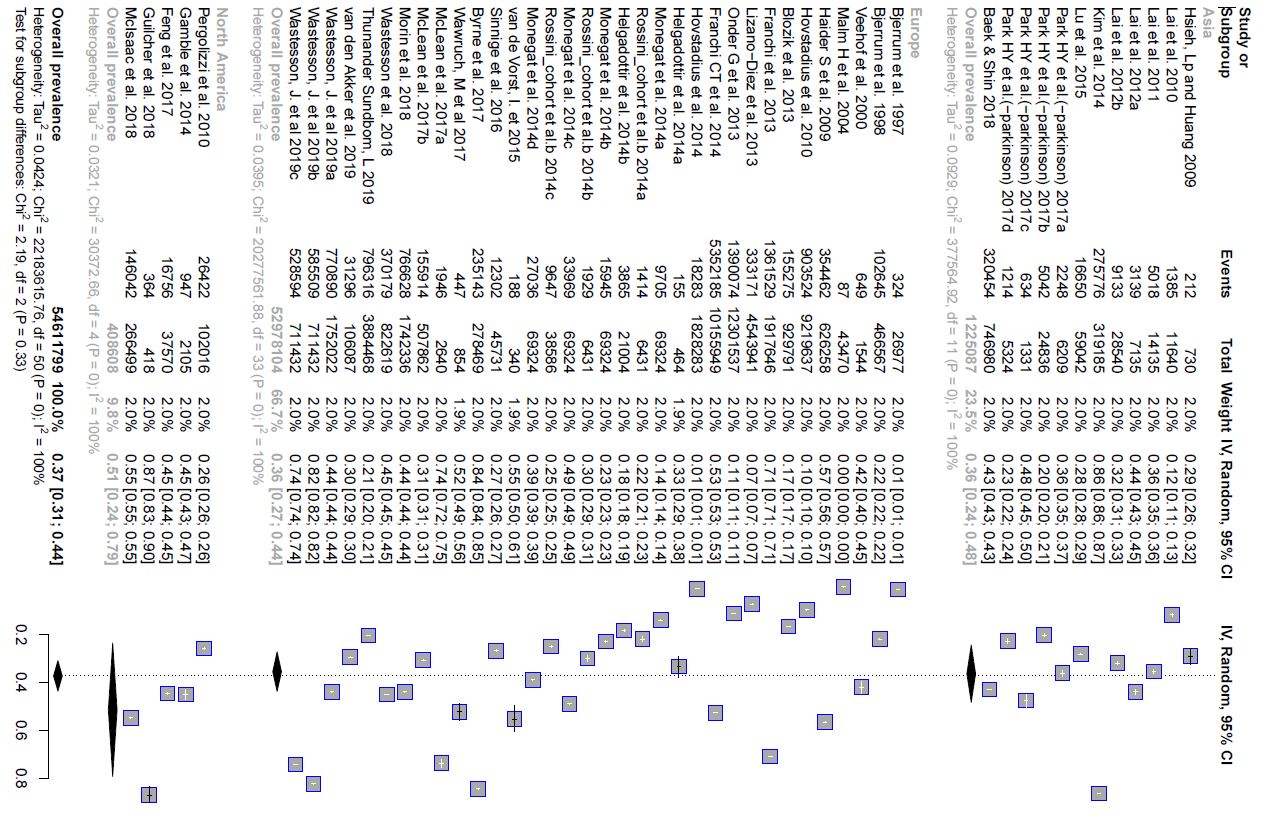

Supplement: Supplementary file 8 — Additional file 8. Prevalence of Polypharmacy Associated with Study Geographical Location. [file 12877_2022_3279_MOESM8_ESM.docx]

## Additional file 9. Prevalence of Polypharmacy Associated with Different Study Designs


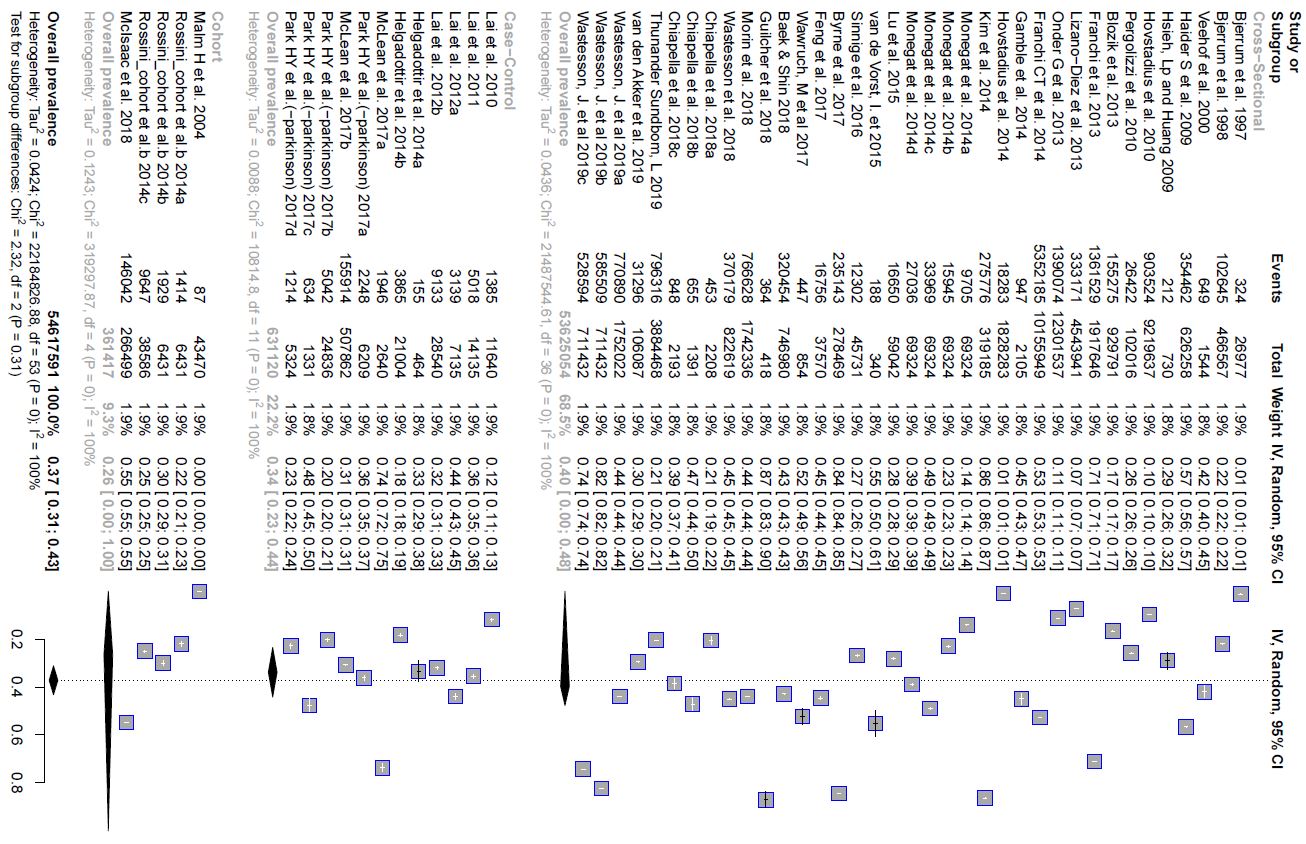

Supplement: Supplementary file 9 — Additional file 9. Prevalence of Polypharmacy Associated with Different Study Designs. [file 12877_2022_3279_MOESM9_ESM.docx]

**Additional file 11.** Funnel Plot of Prevalence of Polypharmacy in Included Studies


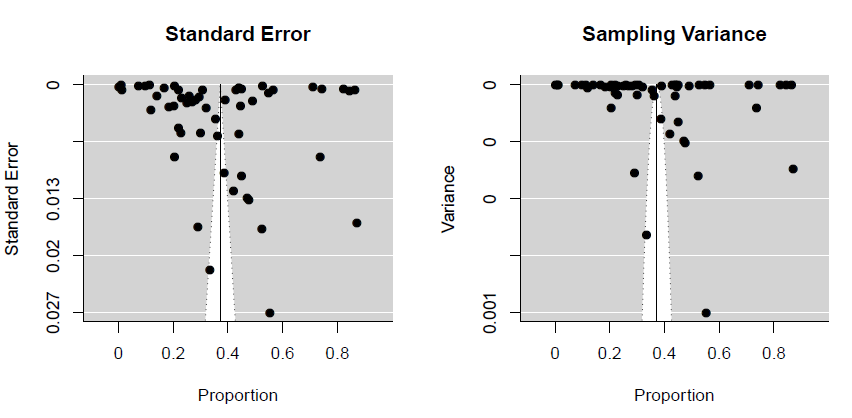

Supplement: Supplementary file 11 — Additional file 11. Funnel Plot of Prevalence of Polypharmacy in Included Studies. [file 12877_2022_3279_MOESM11_ESM.docx]
