# Supplementary material for: Prevalence and factors associated with polypharmacy: a systematic review and Meta-analysis
Source: BMC Geriatr. 2022 Jul 19;22:601. doi: 10.1186/s12877-022-03279-x (PMC9297624; doi:10.1186/s12877-022-03279-x)

## Additional file 5. Prevalence of Polypharmacy Associated with Different Medication Thresholds

##
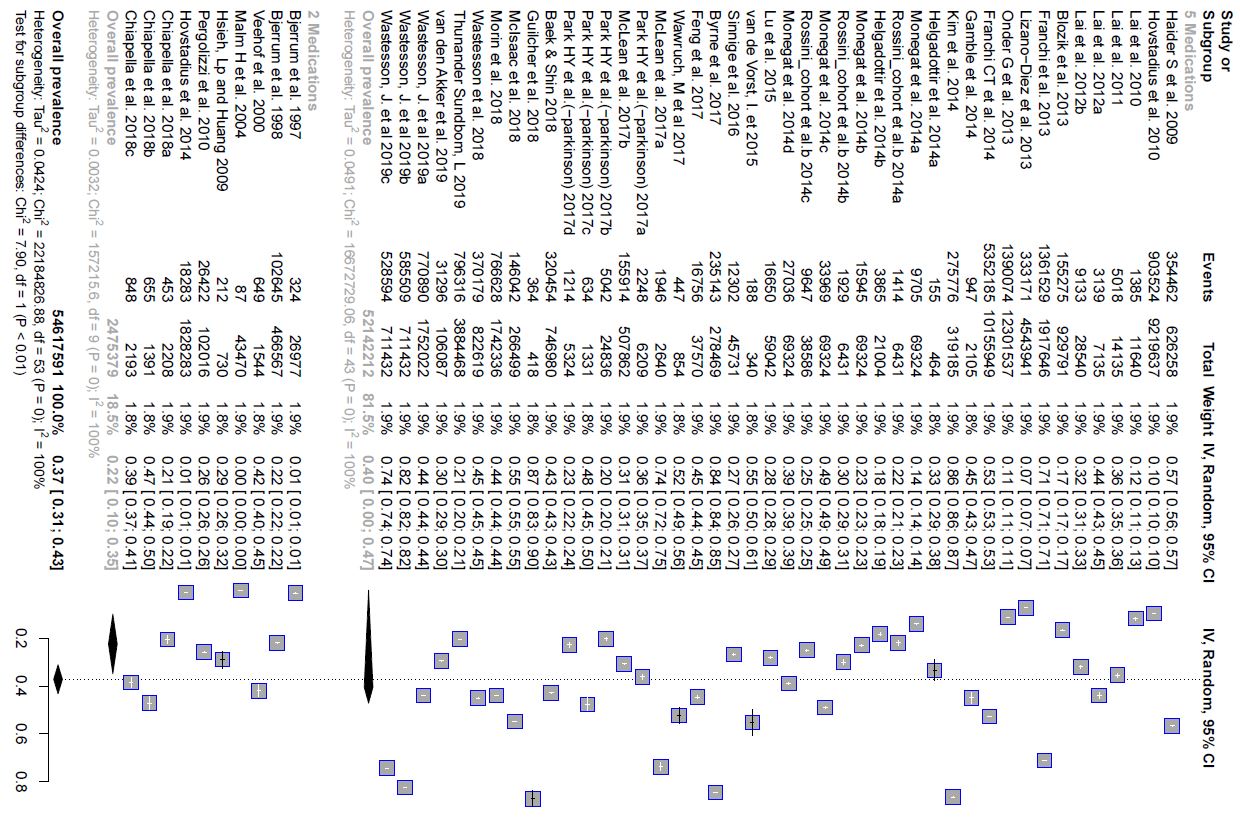

Supplement: Supplementary file 5 — Additional file 5. Prevalence of Polypharmacy Associated with Different Medication Thresholds. [file 12877_2022_3279_MOESM5_ESM.docx]
