# Supplementary material for: Prevalence and factors associated with polypharmacy: a systematic review and Meta-analysis
Source: BMC Geriatr. 2022 Jul 19;22:601. doi: 10.1186/s12877-022-03279-x (PMC9297624; doi:10.1186/s12877-022-03279-x)
Supplement: Supplementary file 10 — Additional file 10. Risk of Bias Summary in Included Studies (n = 106). [file 12877_2022_3279_MOESM10_ESM.docx]

## Additional file 10. Risk of Bias Summary in Included Studies (n=106)

| **Author (year)**  **Study**  **Design** | **Risk of Bias Summary items** | | | | | | | | **Total score** | **Quality** |
| --- | --- | --- | --- | --- | --- | --- | --- | --- | --- | --- |
|  | **Representative of the exposed group** | **Selection of non-exposed group** | **Ascertainment of exposure** | **Absence of outcome at start of study** | **Comparability** | **Assessment of outcome** | **Adequacy of follow up length** | **Adequacy of follow up rate** |  |  |
| Asranna et al. (2018) | ✓ | ✓ | ? | ✓ | ✓ | ? | ✓ | ✓ | 5 | Low |
| Charlton et al. (2011) | ✓ | ✓ | ✓ | ✓ | ? | ✓ | ✓ | ✓ | 7 | High |
| Ekstam & Elmståhl (2016) | ✓ | ✓ | ✓ | ✓ | ✓ | ✓ | ✓ | ✓ | 7 | High |
| Faught et al. (2018) | ✓ | ✓ | ✓ | ✓ | ✓ | ✓ | ✓ | ✓ | 7 | High |
| Hung et al. (2017) | ✓ | ✓ | ? | ✓ | ✓ | ? | ✓ | ✓ | 5 | Low |
| Kadra et al. (2018) | ✓ | ✓ | ✓ | ✓ | ✓ | ✓ | - | ✓ | 6 | Low |
| Kulaga et al. (2011) | ✓ | ✓ | ✓ | ✓ | ✓ | ✓ | ✓ | ✓ | 7 | High |
| Malm et al. (2004) | ✓ | ✓ | ✓ | ✓ | ✓ | ✓ | ✓ | ✓ | 7 | High |
| McIsaac et al. (2018) | ✓ | ✓ | ✓ | ✓ | ✓ | ✓ | ✓ | ✓ | 7 | High |
| Moisan & Grégoire (2010) | ✓ | ✓ | ✓ | ✓ | ✓ | ✓ | ✓ | ✓ | 7 | High |
| Palmsten et al. (2013) | ✓ | ✓ | ✓ | ✓ | ✓ | ✓ | ✓ | ✓ | 7 | High |
| Rossini et al. (2014) | ✓ | ✓ | ✓ | ✓ | ✓ | ✓ | ✓ | ✓ | 8 | High |
| Sanglier et al. (2011) | ✓ | ✓ | ✓ | ✓ | ✓ | ✓ | ✓ | ✓ | 7 | High |
| Dolk et al. (2008) | **-** | **✓** | **-** | **-** | **✓** | **✓** | **-** | **✓** | 6 | Low |
| Frandsen et al. (2014) | **-** | **✓** | **✓** | **✓** | **✓** | **✓** | **✓** | **✓** | 7 | High |
| French et al. (2005) | **-** | **✓** | **-** | **-** | **✓** | **✓** | **-** | **✓** | 4 | Low |
| Helgadóttir et al. (2014) | **-** | **✓** | **✓** | **✓** | **✓** | **✓** | **✓** | **✓** | 7 | High |
| Laflamme et al. (2015) | **-** | **✓** | **✓** | **✓** | **✓** | **✓** | **✓** | **✓** | 8 | High |
| Lai et al. (2012) | **-** | **✓** | **✓** | **✓** | **✓** | **✓** | **✓** | **✓** | 7 | High |
| Lai et al. (2011) | **-** | **✓** | **✓** | **✓** | **✓** | **✓** | **✓** | **✓** | 8 | High |
| Lai et al. (2010) | **-** | **✓** | **✓** | **✓** | **✓** | **✓** | **✓** | **✓** | 7 | High |
| McLean et al. (2017) | **-** | **✓** | **✓** | **✓** | **✓** | **✓** | **✓** | **✓** | 7 | High |
| Park et al. (2017) | **-** | **✓** | **✓** | **✓** | **✓** | **✓** | **✓** | **✓** | 8 | High |
| Park et al. (2017) | **-** | **✓** | **✓** | **✓** | **✓** | **✓** | **✓** | **✓** | 8 | High |
| van Erning et al. (2016) | **✓** | **✓** | **✓** | **✓** | **✓** | **✓** | **✓** | **✓** | 9 | High |
| Yu et al. (2017) | **-** | **✓** | **✓** | **✓** | **✓** | **✓** | **✓** | **✓** | 8 | High |
| Abe et al. (2017) | **✓** | **✓** | **✓** | **✓** | **✓** | **✓** | **✓** | | 8 | High |
| Andrew et al. (2012) | **✓** | **✓** | **✓** | **✓** | **✓** | **✓** | **✓** | | 9 | High |
| Åstrand et al. (2007) | **✓** | **✓** | **✓** | **✓** | **✓** | **✓** | **✓** | | 9 | High |
| Åstrand et al. (2006) | **✓** | **✓** | **✓** | **✓** | **✓** | **✓** | **✓** | | 9 | High |
| Baandrup et al. (2016) | **✓** | **✓** | **✓** | **✓** | **✓** | **✓** | **✓** | | 9 | High |
| Baandrup et al. (2012) | **✓** | **✓** | **✓** | **✓** | **✓** | **✓** | **✓** | | 9 | High |
| Baek & Shin et al. (2018) | **✓** | **✓** | **✓** | **✓** | **✓** | **✓** | **✓** | | 10 | High |
| Bjerrum (1998) | **✓** | **✓** | **✓** | **✓** | **✓** | **✓** | **✓** | | 8 | High |
| Bjerrum et al (1997) | **✓** | **✓** | **✓** | **✓** | **✓** | **✓** | **✓** | | 8 | High |
| Blozik et al. (2013) | **✓** | **✓** | **✓** | **✓** | **✓** | **✓** | **✓** | | 9 | High |
| Broeks et al. (2017) | **✓** | **✓** | **✓** | **✓** | **✓** | **✓** | **✓** | | 8 | High |
| Byrne et al. (2017) | **✓** | **✓** | **✓** | **✓** | **✓** | **✓** | **✓** | | 10 | High |
| Calderón-Larrañaga et al. (2013) | **✓** | **✓** | **✓** | **✓** | **✓** | **✓** | **✓** | | 9 | High |
| Carey et al. (2008) | **✓** | **✓** | **✓** | **✓** | **✓** | **✓** | **✓** | | 10 | High |
| Caughey et al. (2017) | **✓** | **✓** | **✓** | **✓** | **✓** | **✓** | **✓** | | 9 | High |
| Chang et al. (2016) | **✓** | **✓** | **✓** | **✓** | **✓** | **✓** | **✓** | | 9 | High |
| Chiapella et al. (2018) | **✓** | **✓** | **✓** | **✓** | **✓** | **✓** | **✓** | | 8 | High |
| Cho et al. (2018) | **✓** | **✓** | **✓** | **✓** | **✓** | **✓** | **✓** | | 10 | High |
| Constantine et al. (2010) | **✓** | **✓** | **✓** | **✓** | **✓** | **✓** | **✓** | | 9 | High |
| Curkendall et al. (2013) | **✓** | **✓** | **✓** | **✓** | **✓** | **✓** | **✓** | | 9 | High |
| De las Cuevas & Sanz et al. (2004) | **✓** | **✓** | **✓** | **✓** | **✓** | **✓** | **✓** | | 9 | High |
| Degli Esposti et al. (2014) | **✓** | **✓** | **✓** | **✓** | **✓** | **✓** | **✓** | | 8 | High |
| Fano et al. (2014) | **✓** | **✓** | **✓** | **✓** | **✓** | **✓** | **✓** | | 10 | High |
| Feng et al. (2017) | **✓** | **✓** | **✓** | **✓** | **✓** | **✓** | **✓** | | 10 | High |
| Fereshtehnejad et al. (2014) | **✓** | **✓** | **✓** | **✓** | **✓** | **✓** | **✓** | | 9 | High |
| Fontanella et al. (2018) | **✓** | **✓** | **✓** | **✓** | **✓** | **✓** | **✓** | | 10 | High |
| Franchi et al. (2014) | **✓** | **✓** | **✓** | **✓** | **✓** | **✓** | **✓** | | 8 | High |
| Franchi et al. (2013) | **✓** | **✓** | **✓** | **✓** | **✓** | **✓** | **✓** | | 8 | High |
| Gamble et al. (2014) | **✓** | **✓** | **✓** | **✓** | **✓** | **✓** | **✓** | | 9 | High |
| Gaviria et al. (2015) | **✓** | **✓** | **✓** | **✓** | **✓** | **✓** | **✓** | | 10 | High |
| Gidal et al. (2009) | **✓** | **✓** | **✓** | **✓** | **✓** | **✓** | **✓** | | 8 | High |
| Gören et al. (2013) | **✓** | **✓** | **✓** | **✓** | **✓** | **✓** | **✓** | | 9 | High |
| Guidoni et al. (2014) | **✓** | **✓** | **✓** | **✓** | **✓** | **✓** | **✓** | | 9 | High |
| Guilcher et al. (2018) | **✓** | **✓** | **✓** | **✓** | **✓** | **✓** | **✓** | | 10 | High |
| Haider et al. (2009) | **✓** | **✓** | **✓** | **✓** | **✓** | **✓** | **✓** | | 10 | High |
| Hamann et al. (2003) | **✓** | **✓** | **✓** | **✓** | **✓** | **✓** | **✓** | | 7 | High |
| Hoffmann et al. (2011) | **✓** | **✓** | **✓** | **✓** | **✓** | **✓** | **✓** | | 10 | High |
| Horváth et al. (2016) | **✓** | **✓** | **✓** | **✓** | **✓** | **✓** | **✓** | | 10 | High |
| Hovstadius et al. (2014) | **✓** | **✓** | **✓** | **✓** | **✓** | **✓** | **✓** | | 8 | High |
| Hovstadius et al. (2010) | **✓** | **✓** | **✓** | **✓** | **✓** | **✓** | **✓** | | 8 | High |
| Hsieh & Huang et al. (2009) | **✓** | **✓** | **✓** | **✓** | **✓** | **✓** | **✓** | | 9 | High |
| Ivanova et al. (2019) | **✓** | **✓** | **✓** | **✓** | **✓** | **✓** | **✓** | | 10 | High |
| Jaffe & Levine (2003) | **✓** | **✓** | **✓** | **✓** | **✓** | **✓** | **✓** | | 8 | High |
| Kadra et al. (2018) | **✓** | **✓** | **✓** | **✓** | **✓** | **✓** | **✓** | | 10 | High |
| Källén et al. (1989) | **✓** | **✓** | **✓** | **✓** | **✓** | **✓** | **✓** | | 8 | High |
| Kim et al. (2014) | **✓** | **✓** | **✓** | **✓** | **✓** | **✓** | **✓** | | 10 | High |
| Kragh et al. (2011) | **✓** | **✓** | **✓** | **✓** | **✓** | **✓** | **✓** | | 8 | High |
| Landmark et al. (2011) | **✓** | **✓** | **✓** | **✓** | **✓** | **✓** | **✓** | | 9 | High |
| Lin et al. (2015) | **✓** | **✓** | **✓** | **✓** | **✓** | **✓** | **✓** | | 9 | High |
| Lizano-Díez et al. (2013) | **✓** | **✓** | **✓** | **✓** | **✓** | **✓** | **✓** | | 9 | High |
| Lu et al. (2015) | **✓** | **✓** | **✓** | **✓** | **✓** | **✓** | **✓** | | 10 | High |
| Mizokami et al. (2017) | **✓** | **✓** | **✓** | **✓** | **✓** | **✓** | **✓** | | 9 | High |
| Monégat et al. (2014) | **✓** | **✓** | **✓** | **✓** | **✓** | **✓** | **✓** | | 9 | High |
| Morin et al. (2018) | **✓** | **✓** | **✓** | **✓** | **✓** | **✓** | **✓** | | 9 | High |
| Onder et al. (2014) | **✓** | **✓** | **✓** | **✓** | **✓** | **✓** | **✓** | | 9 | High |
| Onishi et al. (2013) | **✓** | **✓** | **✓** | **✓** | **✓** | **✓** | **✓** | | 8 | High |
| Pergolizzi Jr et al. (2011) | **✓** | **✓** | **✓** | **✓** | **✓** | **✓** | **✓** | | 10 | High |
| Pottegård et al. (2014) | **✓** | **✓** | **✓** | **✓** | **✓** | **✓** | **✓** | | 9 | High |
| Preskorn et al. (2005) | **✓** | **✓** | **✓** | **✓** | **✓** | **✓** | **✓** | | 9 | High |
| Ruwald et al. (2013) | **✓** | **✓** | **✓** | **✓** | **✓** | **✓** | **✓** | | 10 | High |
| Salahudeen et al. (2015) | **✓** | **✓** | **✓** | **✓** | **✓** | **✓** | **✓** | | 10 | High |
| Sinnige et al. (2016) | **✓** | **✓** | **✓** | **✓** | **✓** | **✓** | **✓** | | 10 | High |
| Slabaugh et al. (2010) | **✓** | **✓** | **✓** | **✓** | **✓** | **✓** | **✓** | | 9 | High |
| Subesinghe et al. (2018) | **✓** | **✓** | **✓** | **✓** | **✓** | **✓** | **✓** | | 9 | High |
| Suokas et al. (2013) | **✓** | **✓** | **✓** | **✓** | **✓** | **✓** | **✓** | | 9 | High |
| Targownik et al. (2007) | **✓** | **✓** | **✓** | **✓** | **✓** | **✓** | **✓** | | 10 | High |
| Thunander & Hedborg (2019) | **✓** | **✓** | **✓** | **✓** | **✓** | **✓** | **✓** | | 9 | High |
| Tiihonen et al. (2012) | **✓** | **✓** | **✓** | **✓** | **✓** | **✓** | **✓** | | 10 | High |
| van de Vorst et al. (2015) | **✓** | **✓** | **✓** | **✓** | **✓** | **✓** | **✓** | | 9 | High |
| van den Akker et al. (2019) | **✓** | **✓** | **✓** | **✓** | **✓** | **✓** | **✓** | | 9 | High |
| van den Bemt et al. (2016) | **✓** | **✓** | **✓** | **✓** | **✓** | **✓** | **✓** | | 9 | High |
| Veehof et al. (2000) | **✓** | **✓** | **✓** | **✓** | **✓** | **✓** | **✓** | | 10 | High |
| Wang et al. (2014) | **✓** | **✓** | **✓** | **✓** | **✓** | **✓** | **✓** | | 9 | High |
| Wastesson et al. (2018) | **✓** | **✓** | **✓** | **✓** | **✓** | **✓** | **✓** | | 9 | High |
| Wastesson et al. (2019) | **✓** | **✓** | **✓** | **✓** | **✓** | **✓** | **✓** | | 10 | High |
| Wawruch et al. (2017) | **✓** | **✓** | **✓** | **✓** | **✓** | **✓** | **✓** | | 9 | High |
| Weissman (2002) | **✓** | **✓** | **✓** | **✓** | **✓** | **✓** | **✓** | | 8 | High |
| Wong et al. (2013) | **✓** | **✓** | **✓** | **✓** | **✓** | **✓** | **✓** | | 8 | High |
| Xiang et al. (2012) | **✓** | **✓** | **✓** | **✓** | **✓** | **✓** | **✓** | | 9 | High |
| Yeh et al. (2017) | **✓** | **✓** | **✓** | **✓** | **✓** | **✓** | **✓** | | 8 | High |
